# Urine metabolomics phenotyping and urinary biomarker exploratory in mild cognitive impairment and Alzheimer’s disease

**DOI:** 10.3389/fnagi.2023.1273807

**Published:** 2023-12-22

**Authors:** Yuye Wang, Yu Sun, Yu Wang, Shuhong Jia, Yanan Qiao, Zhi Zhou, Wen Shao, Xiangfei Zhang, Jing Guo, Xincheng Song, Xiaoqian Niu, Dantao Peng

**Affiliations:** ^1^China-Japan Friendship Hospital, Chinese Academy of Medical Sciences and Peking Union Medical College, Beijing, China; ^2^Department of Neurology, China-Japan Friendship Hospital, Beijing, China; ^3^Peking University China-Japan Friendship School of Clinical Medicine, Beijing, China; ^4^Department of Neurology, Xijing Hospital, Fourth Military Medical University, Xi’an, China

**Keywords:** urine metabolomics, Alzheimer’s disease, mild cognitive impairment, diagnostic biomarker, machine learning

## Abstract

**Introduction:**

Alzheimer’s disease is a prevalent disease with a heavy global burden and is suggested to be a metabolic disease in the brain in recent years. The metabolome is considered to be the most promising phenotype which reflects changes in genetic, transcript, and protein profiles as well as environmental effects. Aiming to obtain a comprehensive understanding and convenient diagnosis of MCI and AD from another perspective, researchers are working on AD metabolomics. Urine is more convenient which could reflect the change of disease at an earlier stage. Thus, we conducted a cross-sectional study to investigate novel diagnostic panels.

**Methods:**

We first enrolled participants from China-Japan Friendship Hospital from April 2022 to November 2022, collected urine samples and conducted an LC–MS/MS analysis. In parallel, clinical data were collected and clinical examinations were performed. After statistical and bioinformatics analyzes, significant risk factors and differential urinary metabolites were determined. We attempt to investigate diagnostic panels based on machine learning including LASSO and SVM.

**Results:**

Fifty-seven AD patients, 43 MCI patients and 62 CN subjects were enrolled. A total of 2,140 metabolites were identified among which 125 significantly differed between the AD and CN groups, including 46 upregulated ones and 79 downregulated ones. In parallel, there were 93 significant differential metabolites between the MCI and CN groups, including 23 upregulated ones and 70 downregulated ones. AD diagnostic panel (30 metabolites+ age + APOE) achieved an AUC of 0.9575 in the test set while MCI diagnostic panel (45 metabolites+ age + APOE) achieved an AUC of 0.7333 in the test set. Atropine, S-Methyl-L-cysteine-S-oxide, D-Mannose 6-phosphate (M6P), Spiculisporic Acid, N-Acetyl-L-methionine, 13,14-dihydro-15-keto-tetranor Prostaglandin D2, Pyridoxal 5’-Phosphate (PLP) and 17(S)-HpDHA were considered valuable for both AD and MCI diagnosis and defined as hub metabolites. Besides, diagnostic metabolites were weakly correlated with cognitive functions.

**Discussion:**

In conclusion, the procedure is convenient, non-invasive, and useful for diagnosis, which could assist physicians in differentiating AD and MCI from CN. Atropine, M6P and PLP were evidence-based hub metabolites in AD.

## 1 Introduction

Dementia poses a significant global public health challenge. In 2019, the worldwide prevalence of dementia was 57.4 million individuals, with projections indicating a rise to 152.8 million by 2050 (GBD 2019 Dementia Forecasting Collaborators, 2022). Among the different types of dementia, Alzheimer’s disease (AD) is the most prevalent, accounting for approximately 60 to 80% of cases (Alzheimers Dement, 2020). In China’s older population aged 60 and above, it is estimated that 15.07 million individuals are living with dementia, while 9.83 million have specifically been diagnosed with AD (Jia et al., 2020). These data highlight the substantial burden on China’s society and economy that cannot be overlooked. Mild cognitive impairment (MCI), considered as a pre-dementia stage with clinical symptoms, represents a continuum of cognitive decline where individuals experience mild cognitive deficits without requiring assistance for daily activities. Early identification of MCI can serve as an indication of increased risk for developing AD, and early comprehensive interventions hold the potential to delay or prevent the progression from MCI to dementia (Langa and Levine, 2014).

Recently, studies have revealed that several pathophysiological processes associated with insulin resistance are common to both AD and diabetes mellitus (Kandimalla et al., 2017; Hamze et al., 2022; Michailidis et al., 2022). Amylin, tau protein, and beta-amyloid may gather in the brains of people who suffered type 2 diabetes mellitus and AD (Michailidis et al., 2022). Based on the evidence, many scholars suggested that AD is type 3 diabetes (Kandimalla et al., 2017; Nguyen et al., 2020; Hamze et al., 2022; Michailidis et al., 2022). The underlying cause of amyloid proteinopathy and its related neurodegeneration in AD is considered as metabolism dysfunction (Poddar et al., 2021). In other words, AD is a metabolic disease in the brain. Thus, identifying metabolic alterations during AD disease trajectory and their connection to clinical phenotypes contributed to a deep understanding of AD and further provided a powerful basis for drug and biomarker discovery (Toledo et al., 2017).

Many studies have been conducted on the metabolome, a collection of small-molecule chemical elements involved in metabolism, to identify and predict biomarkers for disease, and further, for the discovery of active drivers of biological processes (Rinschen et al., 2019). The metabolome is thought to be the most promising phenotype (Schrimpe-Rutledge et al., 2016) which reflects alterations in gene, transcript, and protein profiles and environmental effects (Wilkins and Trushina, 2017). In AD application, CSF (Kaddurah-Daouk et al., 2013; Muguruma et al., 2018) or blood (Wang et al., 2014; Tynkkynen et al., 2018) metabolomics revealed several diagnostic panels and involved pathways. However, homeostatic processes might mitigate alterations in CSF and blood brought on by brain disorders. Urine, non-invasive and readily available bio-fluid, is not dependent on homeostatic systems, which can reflect a lot of variations that could indicate how the body is functioning (Wu and Gao, 2015). The probable application of urinary biomarkers for brain diseases is usually disregarded. Actually, urine could be applicated as biomarkers for brain diseases (An and Gao, 2015) and neurodegenerative diseases (Seol et al., 2020), including AD. The role of urinary metabolomics remained to be illustrated.

In this study, we initially enrolled AD patients, MCI patients and cognitive normal (CN) participants. Then we collected urine samples and the urine were undergone an ultra-high-performance liquid chromatography coupled with mass spectrometry (UHPLC–MS/MS) test. We aim to identify novel diagnostic panels for early diagnosis of MCI and AD based on urine metabolomics and machine learning and provide a basis for the discovery of the active role of metabolites in AD. The study protocol was approved by the China-Japan Friendship Hospital ethics committee and institutions (Ethics ID: 2020-31-Y06-32). Consent forms were obtained from all participants.

## 2 Methods

### 2.1 Subjects enrollment

A total of 162 participants, over 50 years old, including 57 AD patients, 43 MCI patients and 62 CN subjects were included in this cross-sectional study. All participants were enrolled in China-Japan Friendship Hospital from April 2022 to November 2022. Apolipoprotein E (APOE) genotype testing, a battery of cognitive tests, and medical history gathering were all performed on each participant. The majority of participants underwent quantitative electroencephalography (qEEG) and Magnetic Resonance Imaging (MRI).

AD is clinically diagnosed with the 2011 National Institute on Aging-Alzheimer’s Association (NIA-AA) criteria (McKhann et al., 2011). MCI is also defined by the 2011 NIA-AA diagnostic criteria (Albert et al., 2011). CN controls were those who performed normally on the standardized neuropsychological tests and with or without cognitive complaints or concerns during the structured interview.

Listed below are exclusion criteria: (1) Cognitive dysfunction caused by severe psychiatric disorders or mental retardation, (2) Cognitive decline resulting from other nervous diseases, such as trauma, stroke, tumor, Parkinsonism, encephalitis or epilepsy or other types of dementia, such as vascular dementia (VaD), frontotemporal dementia (FTD), and Lewy body dementia (LBD), (3) Cognitive decline resulting from diseases of other systems such as severe anemia and thyroid disorders, (4) A history of malignant tumor, severe diseases, or other conditions affecting the urinary system, (5) A refusal to participate during neuropsychological testing; or incomplete clinical data.

The neuropsychological test battery included measures of global cognition such as Mini-Mental State Examination (MMSE) and Montreal Cognitive Assessment (MoCA) and cognitive performance in the domains of memory, executive function, attention, language and visuospatial ability. Activity of Daily Living Scale (ADL) was used for accessing the function ability during daily life. The specific scales have been applied in clinical practice and published in previous articles from our team (Qiao et al., 2022).

### 2.2 Metabolites extraction

The urine samples (200 μL) were placed in the centrifuge tubes and re-suspended with prechilled 80% methanol by well vortex. The samples were then centrifuged at 15,000 g for 20 min at 4°C after being incubated on ice for 5 min. A portion of the supernatant was diluted with LC–MS grade water to a final concentration that contained 53% methanol. The samples were then moved to a brand-new centrifuge tube and centrifuged there for 20 min at a speed of 15,000 g at 4°C. The supernatant was then added to the analysis of the LC–MS/MS system (Want et al., 2006; Barri and Dragsted, 2013).

### 2.3 UHPLC–MS/MS analysis

In Novogene Co., Ltd. (Beijing, China), UHPLC–MS/MS analyzes were carried out utilizing a Vanquish UHPLC system (Thermo Fisher, Germany) paired with an Orbitrap Q Exactive™HF mass spectrometer (Thermo Fisher, Germany). A 17-min linear gradient was used to inject samples onto a HypesilGoldcolumn (100 × 2.1 mm, 1.9 ) at a flow rate of 0.2 mL/min. Eluents A (0.1% formic acid (FA) in water) and B (methanol) were used in the positive polarity mode. Eluents A (5 mM ammonium acetate, pH 9.0) and B (Methanol) were used in the negative polarity mode. The following settings were made for the solvent gradient: 2% B, 1.5 min; 2–85% B, 3 min; 85–100% B, 10 min; 100–2% B, 10.1 min; 2% B, 12 min. With a spray voltage of 3.5 kV, capillary temperature of 320°C, sheath gas flow rate of 35 psi, aux gas flow rate of 10 L/min, S-lens RF level of 60, and aux gas heater temperature of 350°C, the QExactive^™^HF mass spectrometer was operated in positive/negative polarity mode.

### 2.4 Data processing and metabolite identification

Peak alignment, peak selection, and quantification for each metabolite were carried out using Compound Discoverer 3.1 (CD3.1, Thermo Fisher) to handle the raw data files produced by UHPLC–MS/MS. The following primary parameters were set: 0.2 min for the retention time tolerance; 5 ppm for actual mass tolerance; 30% for the signal intensity tolerance; 3 for the signal/noise ratio; and minimum intensity, etc. Peak intensities were then normalized to the total spectral intensity. The molecular formula was predicted using the normalized data based on additive ions, molecular ion peaks, and fragment ions. The peaks were then compared with the MassList, mzVault, and mzCloud databases to generate accurate qualitative and relative quantitative findings. When data were not normally distributed, standardize using the following method to produce relative peak areas: sample raw quantitation value/(The sum of sample metabolite quantitation value/The sum of QC1 sample metabolite quantitation value); Finally, the findings of the relative quantification and metabolite identification were determined after molecules with relative peak areas in QC samples with CVs more than 30% were eliminated. The Kyoto Encyclopedia of Genes and Genomes (KEGG)^1^, Human Metabolome Database (HMDB),^2^ and Lipid metabolites and pathways strategy (LIPIDMaps)^3^ databases were used to annotate these metabolites.

### 2.5 Statistical analysis and bioinformatics analysis

The statistical software R (version 3.4.3) and Python (version 2.7.6) were used to conduct the statistical analysis. Partial least squares discriminant analysis (PLS-DA) were performed at metaX (Wen et al., 2017). We used *t*-test to determine the statistical significance (*p*-value). The metabolites with Variable Importance in the Projection (VIP) > 1 and *value of p*<0.05 and fold change (FC) ≥ 1.2 or FC ≤ 0.833 were regarded as differential metabolites. The functions of these metabolites and metabolic pathways were researched using the KEGG database. SPSS 23.0 was used for statistical analysis. The Shapiro–Wilk test was used to examine the normality of quantitative data. Mean (x ± s) was used for the description of normal data while non-normal data used median (P25, P75). Analysis of Variance (ANOVA) was used for normal data mean comparison while the Kruskal-Wallis H test was utilized for non-normal data distribution comparison. For *post hoc* comparisons, value of ps were Bonferroni-corrected. Besides, Pearson’s chi-square test or Fisher’s exact probability were used for comparision of the proportions of categorical variables. Statistical significance was defined as a two-tailed value of *p*<0.05.

### 2.6 Machine learning

Machine learning was utilized to identify the optimal multivariate signatures, which took both metabolites and demographic data (age and APOE ε4 status) as input parameters, in order to discriminate AD from CN and MCI from CN. The classifier was made up of feature selection and classifiers (Shi et al., 2019). The dataset was separated into a training set (0.7) and a test set (0.3). The “n” top input variables with the lowest mean square error (MSE) that best distinguished AD or MCI diagnostic groups were chosen using the least absolute shrinkage and selection operator (LASSO). Support vector machine (SVM) classifiers were constructed in order to anticipate the outcome under 10-fold cross-validation on top of these “n” features. The kernel functions of linear, polynomial, radial, and sigmoid were contrasted. When evaluating the model in the test set, accuracy and area under the curve (AUC) (Receiver Operating Characteristic, ROC curve) were applied to assess the diagnostic value.

## 3 Results

### 3.1 Metabolites identification and differential metabolites

**Figure 1:**
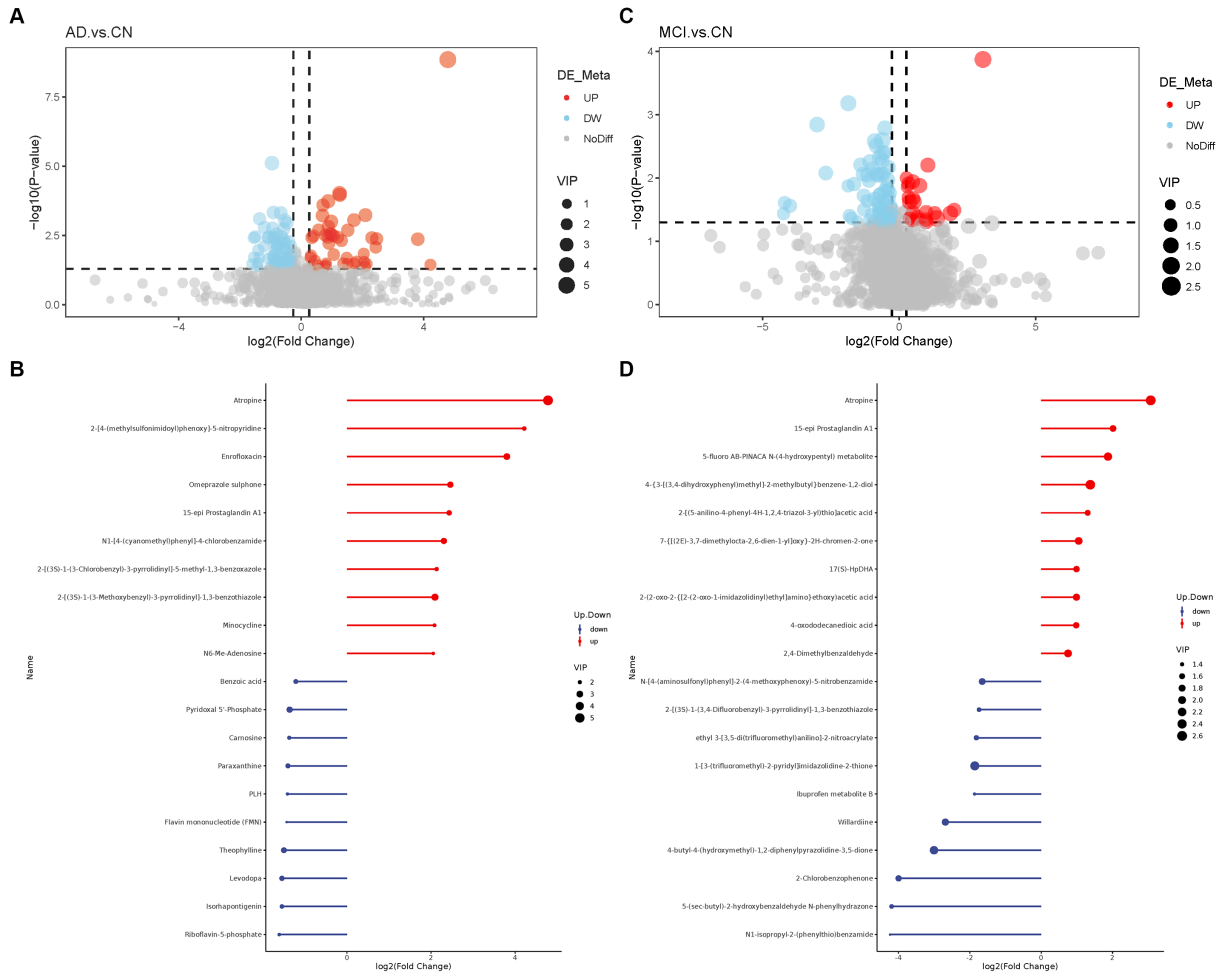
Volcano plots of differential metabolites and stem plots of the top 20 differential metabolites ranged by fold change. **(A)** Volcano plot showed the distribution of all metabolites between AD and CN. **(B)** Stem plot of top 20 differential metabolites in AD-CN group. **(C)** Volcano plot showed the distribution of all metabolites between MCI and CN. **(D)** Stem plot of top 20 differential metabolites in MCI-CN group. Red indicates upregulation and blue indicates downregulation. The size of the circle indicates the VIP value.

Basic information and clinical characteristics of enrolled participants were included in Supplementary material. A total of 2,140 metabolites were identified. Compared to the CN group, significantly differential metabolites were filtered in the AD group and MCI group by setting VIP > 1.0, FC > 1.2 or < 0.833, and *value of p*<0.5. The expression of the differential metabolites in AD group was displayed as a volcano plot and the top 10 regulated metabolites either upregulated or downregulated were displayed in a stem plot (Figures 1A,B) while the expression of the differential metabolites in MCI group was shown in Figures 1C,D. There were 125 significantly differential metabolites between the AD and CN groups, including 46 upregulated ones and 79 downregulated ones. In parallel, there were 93 significantly differential metabolites between the MCI and CN groups, including 23 upregulated ones and 70 downregulated ones. 23 metabolites were significantly regulated in both AD and MCI group, including 6 upregulated ones and 17 downregulated ones. Atropine was the most upregulated metabolite in both AD and MCI group while riboflavin-5-phosphate and N1-isopropyl-2-(phenylthio)benzamide was the most downregulated metabolite in AD and MCI, respectively. All differential metabolites were shown in a heatmap in Supplementary Figure S1. Z-scores of the top 30 differential metabolites were shown in Supplementary Figure S2.

### 3.2 Correlation between metabolites

**Figure 2:**
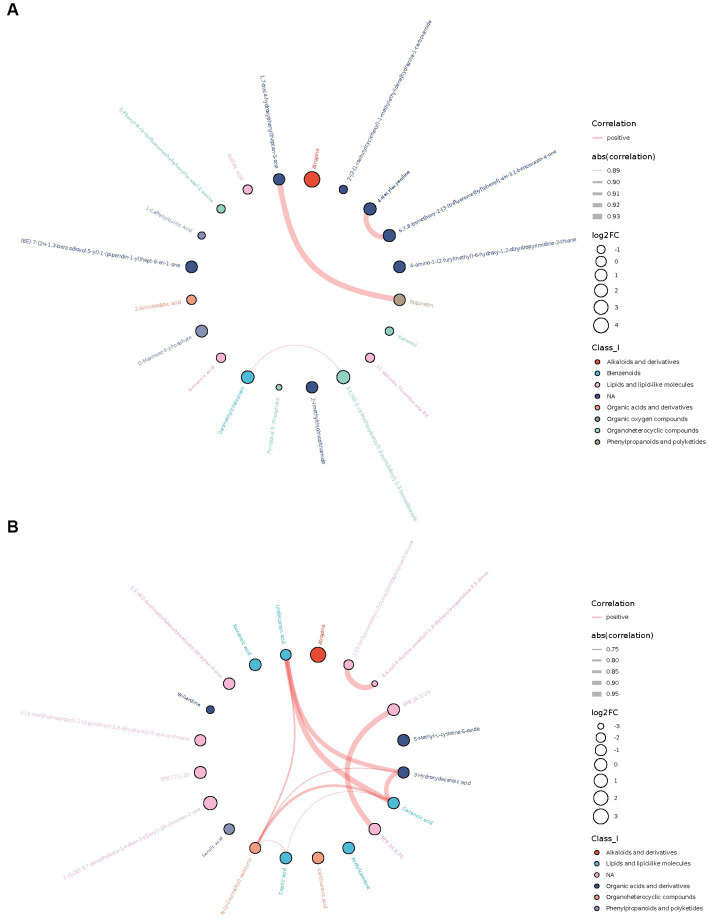
Chord plots showing the relationship between the top 20 differential metabolites ranged by correlation *p*-value. **(A)** Correlation between top 20 differential metabolites in AD-CN group. **(B)** Correlation between top 20 differential metabolites in MCI-CN group. The red line indicates a positive correlation. The width of the line indicates the absolute value of the correlation coefficient. The size of the circle indicates log2 fold change. The colors of the circles indicate the classification of metabolites.

Among the top 20 differential metabolites ranged by value of p, many metabolites were correlated with each other, most positively. A strong correlation (r > 0.7) between metabolites was shown in the chord diagram (Figure 2). In AD-CN group, robinetin was found to be positively correlated with 1,7-bis(4-hydroxyphenyl) heptan-3-one, 4-hexyloxyaniline was found to be positively correlated with 6,7,8-trimethoxy-2-[3-(trifluoromethyl) phenyl]-4H-3,1-benzoxazin-4-one and desmethylcitalopram was found to be positively correlated with 2-[(3S)-1-(3-Methoxybenzyl)-3-pyrrolidinyl]-1,3-benzothiazole. In MCI-CN group, 3-hydroxydecanoic acid, decanoic acid, undecanoic acid and N-(p-Coumaroyl) serotonin were found to be positively correlated with each other. Decanoic acid, N-(p-Coumaroyl) serotonin and capric acid were also found to be positively correlated with each other. There was also strong positive relationship between metabolites which could not be categorized. The overall correlation heatmap was shown in Supplementary Figure S3 when the red indicated a positive relationship and the blue indicated a negative relationship.

### 3.3 Enrichment results of KEGG analysis

**Figure 3:**
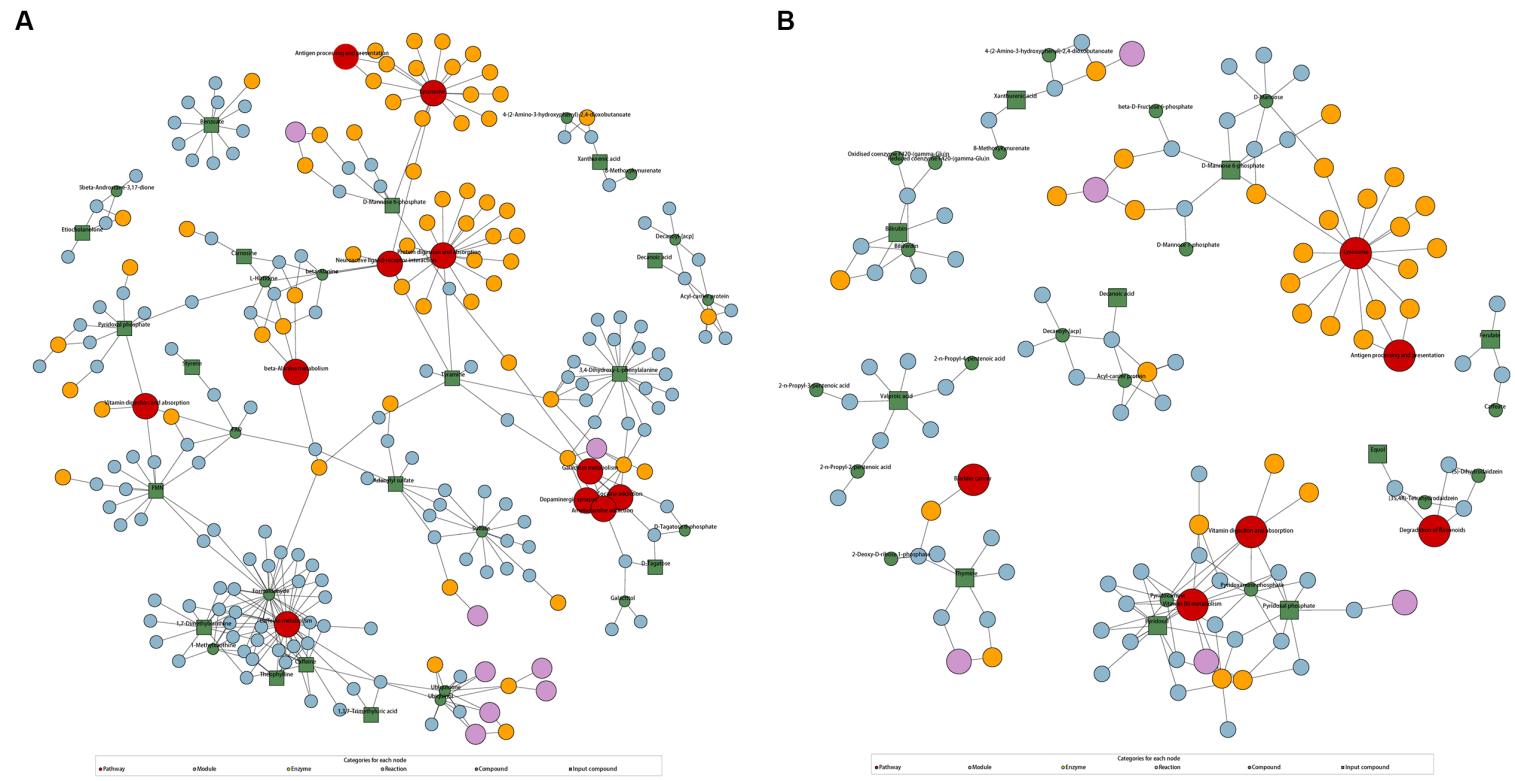
KEGG regulation map enriched by differential metabolites. **(A)** KEGG regulation map in AD-CN group. **(B)** KEGG regulation map in MCI-CN group. The red circle indicates a metabolism pathway. The purple circle indicates a module of a class of metabolites. The yellow circle indicates an enzyme related to a certain substance. The blue circle indicates interactions between chemical substances. The green circle indicates background substance in a metabolism pathway. The green square indicates input differential metabolites.

According to KEGG enrichment analysis results, caffeine metabolism was enriched in AD-CN group (*p* < 0.05). In parallel, vitamin B6 metabolism and fructose and mannose metabolism pathway were enriched in MCI-CN group (p < 0.05). The relative pathways were shown in Supplementary Figure S4. The metabolic network showed the relationship among compounds, pathways, modules, enzymes and reactions to facilitate the presentation of the overall metabolic response. In AD-CN group, 32 compounds were mapped with the KEGG database while 17 compounds were differential metabolites as indicated by green squares. Neuroactive ligand-receptor interaction, galactose metabolism, dopaminergic synapse and other 8 metabolism pathways were enriched as shown in Figure 3A. In MCI-CN group, 29 compounds were mapped with the KEGG database while 10 compounds were differential metabolites as indicated by green squares. Degradation of flavonoids and vitamin B6 metabolism, and other 4 metabolism pathways were enriched as shown in Figure 3B. Antigen processing and presentation, vitamin digestion and absorption and lysosome were enriched in both AD-CN and MCI-CN group.

### 3.4 Identification of novel diagnostic panel

**Figure 4:**
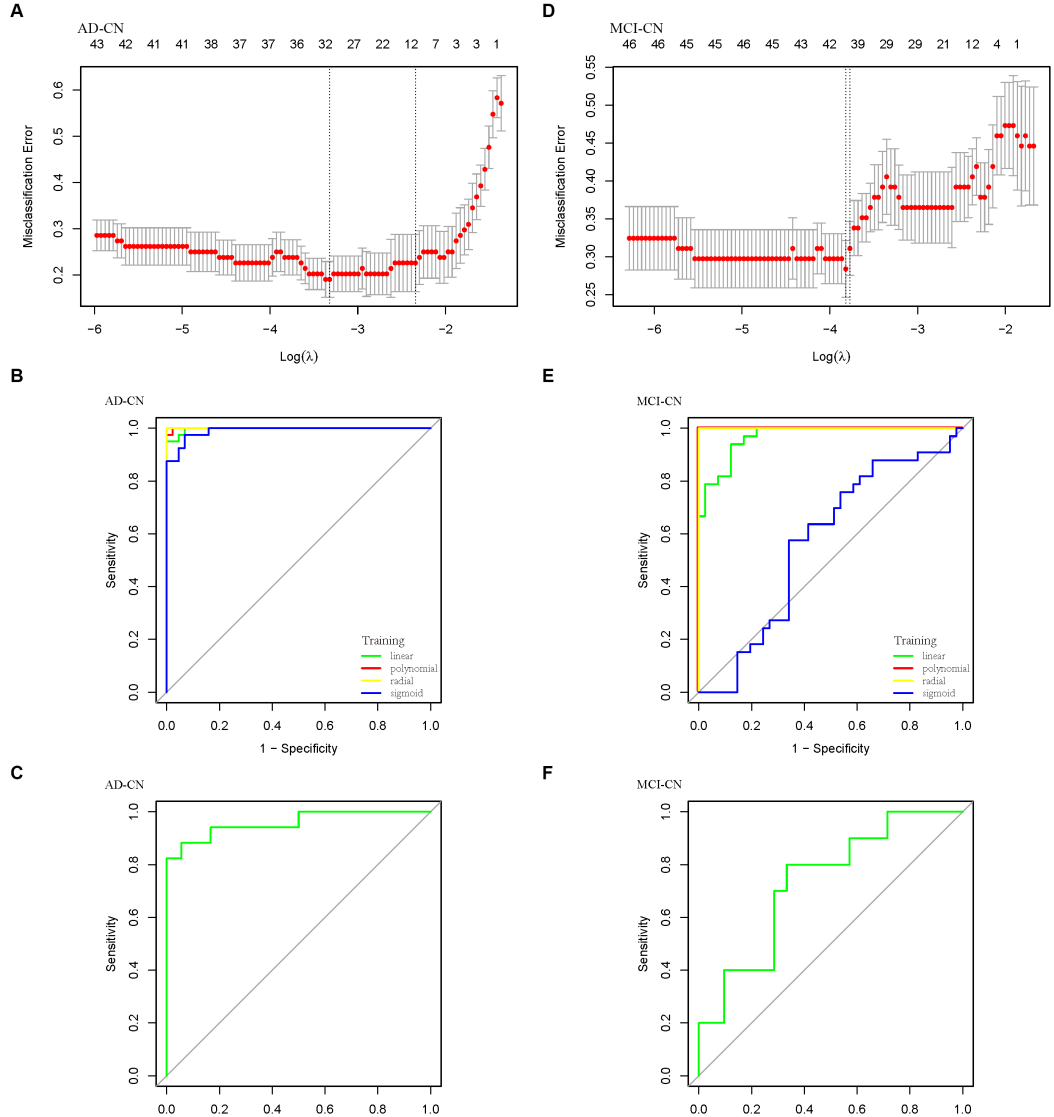
Diagnostic panel selection and ROC curve constructed by LASSO and SVM. **(A)** LASSO model for variable selection in AD-CN group. **(B)** ROC curve for AD diagnosis in the training set. **(C)** ROC curve for AD diagnosis in the test set. **(D)** LASSO model for variable selection in MCI-CN group. **(E)** ROC curve for MCI diagnosis in the training set. **(F)** ROC curve for MCI diagnosis in the test set.

Based on previous analysis, we extracted differential metabolites plus age and APOE ε4 status to construct the LASSO model. Based on LASSO results, we built SVM classifiers with 10-fold cross-validation to investigate the ideal multivariate signatures that distinguished AD from CN. After training in training sets, we compared the results of test sets using different kernel functions in SVM. For AD-CN model, 30 metabolites, age and APOE ε4 status were identified when MSE reached minimum with the value of lambda (min) equaling 0.03614 (Figure 4A). The linear kernel function achieved the highest predictive value with an accuracy of 0.9143 in AD-CN group. Figures 4B,C showed the ROC curves in training set and test set in AD-CN group. Similarly, for MCI-CN model, 45 metabolites, age and APOE ε4 status were identified when MSE reached minimum with the value of lambda (min) equaling to 0.02197 (Figure 4D). Linear kernel function achieved the highest predictive value with an accuracy of 0.6452 in MCI-CN group while Figures 4E,F showed the ROC curves in MCI-CN group. The optimal model achieved an AUC of 0.9575 in AD-CN group and an AUC of 0.7333 in MCI-CN group in test sets. The specific metabolites included in the diagnostic panel were shown in Supplementary Table S3. The evaluation of diagnostic models was shown in Supplementary Table S4.

**Figure 5:**
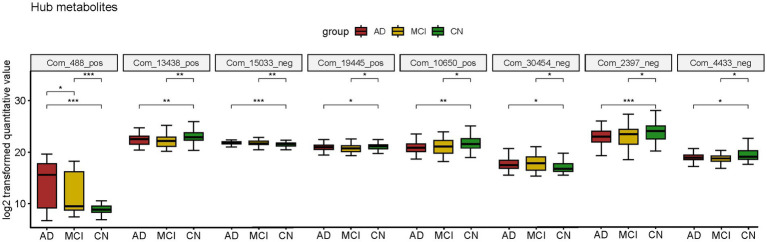
Boxplot of hub metabolites intersected by two diagnostic panels constructed by LASSO model; *p < 0.05; **p < 0.01; ***p < 0.001.

Atropine, S-Methyl-L-cysteine-S-oxide, D-Mannose 6-phosphate (M6P), Spiculisporic Acid, N-Acetyl-L-methionine, 13,14-dihydro-15-keto-tetranor Prostaglandin D2, Pyridoxal 5’-Phosphate (PLP) and 17(S)-HpDHA were considered valuable for both AD and MCI diagnosis. They were also differential metabolites for both AD-CN and MCI-CN group and were defined as hub metabolites. The boxplots showed the log2 transformed quantitative value (Figure 5).

### 3.5 Relationship among hub metabolites and cognitive functions

**Figure 6:**
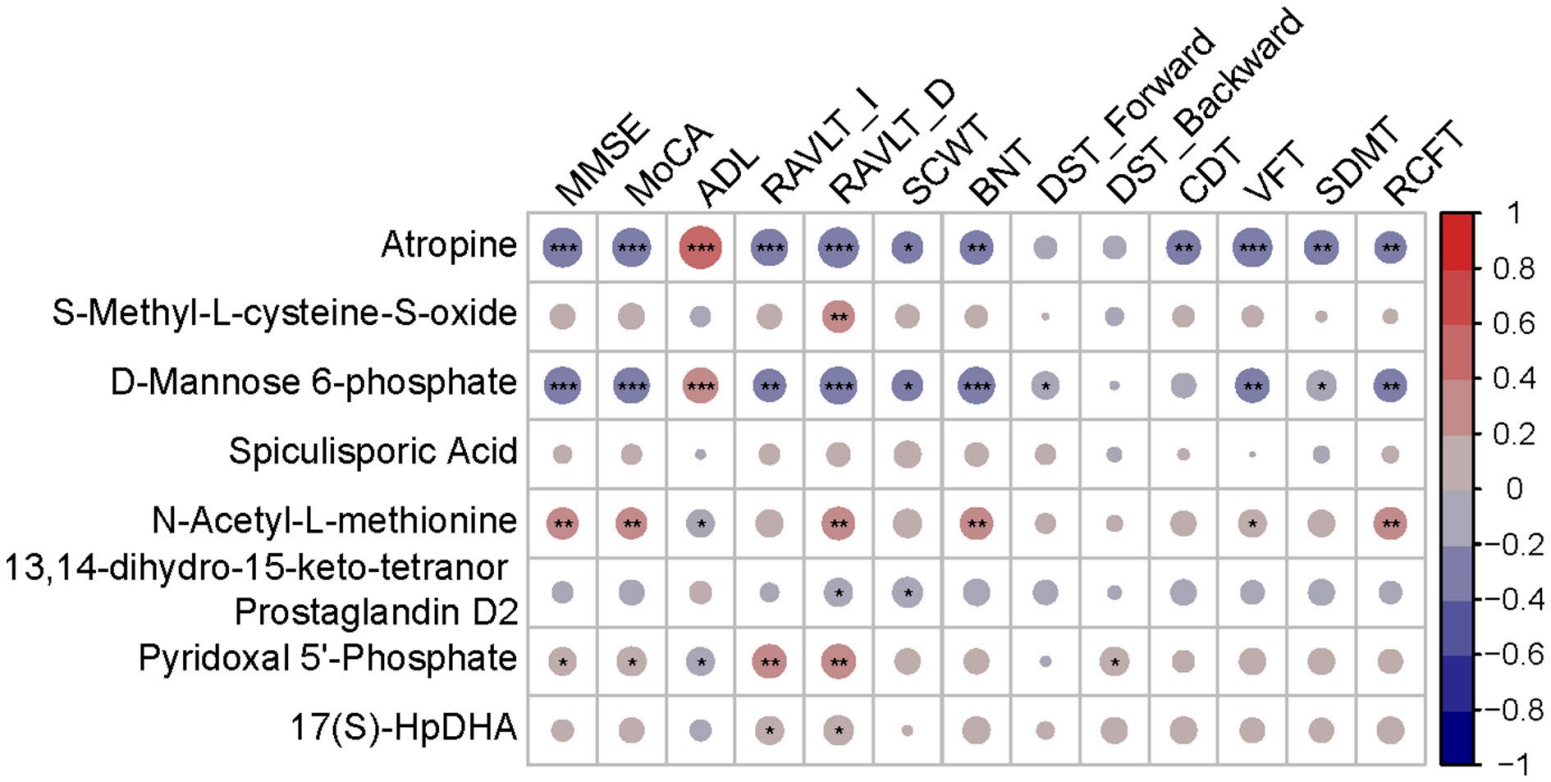
Correlation heatmap between diagnostic metabolites and cognition tests. Red indicates a positive correlation. Blue indicates a negative correlation; *p < 0.05; **p < 0.01; ***p < 0.001.

Hub metabolites were found to be correlated with cognitive tests, although most weakly (Figure 6). Significant labels were shown on the dots. Among 8 diagnostic metabolites, atropine, M6P and N-Acetyl-L-methionine were significantly correlated with more than half of cognitive tests while S-Methyl-L-cysteine-S-oxide, 13,14-dihydro-15-keto-tetranor Prostaglandin D2, PLP and 17(S)-HpDHA were significantly correlated with less than half cognitive tests. Nevertheless, none of the correlations between Spiculisporic Acid and cognitive domains reach significance. The relative ρ and p were shown in Supplementary Table S5 and scatter dot plots were shown in Supplementary Figures S5–S11.

## 4 Discussion

In this research, we first enrolled 57 AD patients, 43 MCI patients and 62 CN subjects from China-Japan Friendship Hospital from April 2022 to November 2022, collected urine samples and conducted an UHPLC–MS/MS analysis. Age and APOE 4 status were notable risk variables, in line with earlier findings. Most clinical indicators varied across the groups. Then, we reported the identified urine metabolites and conducted differential analysis. After filtering out differential metabolites, correlation analysis between metabolites and kegg enrichment were performed. Next, we attempted to figure out novel diagnostic panels based on LASSO and SVM models. AD diagnostic panel (30 metabolites+ age + APOE) achieved an AUC of 0.9575 in the test set while MCI diagnostic panel (45 metabolites+ age + APOE) achieved an AUC of 0.7333 in the test set. Diagnostic metabolites that appeared in both AD-CN panel and MCI-CN panel were defined as hub metabolites. Finally, we conducted a correlation analysis among hub metabolites and clinical indicators and found that diagnostic metabolites were weakly correlated with cognitive functions.

In previously published articles, several studies reported the role of urine metabolites in AD. 17(RS)-10-epi-SC-Δ15-11-dihomo-IsoF, prostaglandin E2, neuroprostanes, isoprostanes, isofurans (Garcia-Blanco et al., 2018), γ-aminobutyric acid, glutamate (Zhou et al., 2020), xanthurenic acid, kynurenic acid, serotonin, 5-hydroxyindoleacetic acid and tryptophan (Whiley et al., 2021) were found to differentially expressed in disease group and control group. Moreover, several studies performed diagnostic tests, either. Kurbatova et al. (2020) built a random forests model with 1,542 metabolites along with age, sex and study site. The model was trained in AD and CN samples with an AUC of 0.99 and tested in stable and converted MCI samples which resulted in an AUC of 0.88. Zhang et al. (2022) identified 19 differential metabolites as diagnostic panel and achieved an AUC of 0.976. Similar to our methods, Yilmaz et al. (2020) used LASSO and SVM as well as logistic regression to construct diagnostic models (AD-CN, AD-MCI, MCI-CN) and achieved 3 AUC more than 0.9. Capryloylglycine (Zhang et al., 2022), caffeine and paraxanthine (Watanabe et al., 2021) were also found to be downregulated in AD samples which were consistent with our results. Unfortunately, our results failed to match with previous diagnostic metabolites.

Among hub metabolites, atropine, a cholinergic antagonist, could interfere with cholinergic dysfunction in AD (Ma et al., 2013; Muramatsu et al., 2019; Alcantara-Gonzalez et al., 2021). M6P glycosylation is an important post-translational modification and is involved in several other biological processes. Aberrant M6P modifications were implicated in AD (Huang et al., 2019) which might be related to excretion values. PLP, the active form of vitamin B6, was involved in neurotransmitter biosynthesis which was related to AD (Parra et al., 2018) and was suggested to improve learning and memory capabilities in Aβ_25–35_-injected mice (Choi et al., 2022). However, we failed to find studies investigating the role of S-Methyl-L-cysteine-S-oxide, Spiculisporic Acid, N-Acetyl-L-methionine, 13,14-dihydro-15-keto-tetranor Prostaglandin D2, or 17(S)-HpDHA in AD.

As for the KEGG pathway, a protective role of caffeine in AD was suggested (Larsson et al., 2022). Caffeine metabolism pathway was reported to be associated with AD (Watanabe et al., 2021; Dong et al., 2022; Siokas et al., 2022) which was consistent with our result. In healthy older adults, lower vitamin B_6_, as measured by PLP, was associated with a higher risk of accelerated cognitive decline (Hughes et al., 2017). In MCI patients, supplementing B-vitamin could reduce whole brain atrophy rate (Wu et al., 2021) or enhance cognitive function, as indicated by MMSE (Lee et al., 2016). Besides, vitamin B6 intake was positively associated with BNT in MCI patients (Kim et al., 2014). The role of vitamin B6 supported the significance of vitamin B6 metabolism pathway in MCI-CN group.

Our findings should be released with concern due to several limitations. On one side, the patients came from a single site. We lacked real-world research from multiple hospitals and communities. Whether the findings can apply to other populations, more research is required. In another, no *in vivo* or *in vitro* experiments were conducted to investigate the function of metabolites and mechanisms of the diagnostic metabolites described in this study that participate in AD pathophysiological processes. Besides, the metabolic profiling was associated with dietary pattern (Nik Mohd Fakhruddin et al., 2020) or other factors. We failed to match all the confounding factors. Thus, some of these results may be coincidental.

In conclusion, we performed proteomics analysis based on UHPLC–MS/MS using urine samples from 57 AD patients, 43 MCI patients and 62 CN subjects. After multiple traditional statistical analyzes and bioinformatics analyzes, we identified a novel AD diagnostic panel that included 30 metabolites, age and APOE ε4 and an MCI diagnostic panel that included 45 metabolites, age and APOE ε4. The urine diagnostic panel could help clinicians differentiate AD and MCI from CN, the method of which is convenient, non-invasive, and valuable for diagnosis. Atropine, M6P and PLP were evidence-based hub metabolites in AD while the role of S-Methyl-L-cysteine-S-oxide, Spiculisporic Acid, N-Acetyl-L-methionine, 13,14-dihydro-15-keto-tetranor Prostaglandin D2, or 17(S)-HpDHA in AD need to be investigated.

## Data availability statement

The raw data generated in this paper have been deposited at the National Genomics Data Center (NGDC) OMIX database (OMIX ID: OMIX005414, https://ngdc.cncb.ac.cn/omix/release/OMIX005414).

## Ethics statement

The studies involving humans were approved by China-Japan Friendship Hospital ethics committee and institutions China-Japan Friendship Hospital ethics committee and institutions (Ethics ID: 2020-31-Y06-32). The studies were conducted in accordance with the local legislation and institutional requirements. The participants provided their written informed consent to participate in this study.

## Author contributions

YuyW: Data curation, Formal analysis, Investigation, Methodology, Software, Visualization, Writing – original draft. YS: Writing – review & editing, Supervision, Validation. YuW: Resources, Writing – review & editing. SJ: Resources, Writing – review & editing. YQ: Resources, Writing – review & editing. ZZ: Resources, Writing – review & editing. WS: Resources, Writing – review & editing. XZ: Methodology, Writing – review & editing. JG: Methodology, Writing – review & editing. XS: Data curation, Writing – review & editing. XN: Data curation, Writing – review & editing. DP: Conceptualization, Funding acquisition, Resources, Supervision, Writing – review & editing.

## Footnotes

^1^ https://www.genome.jp/kegg/pathway.html

^2^ https://hmdb.ca/metabolites

^3^ http://www.lipidmaps.org/
