# Supplementary material for: Urine metabolomics phenotyping and urinary biomarker exploratory in mild cognitive impairment and Alzheimer’s disease
Source: Front Aging Neurosci. 2023 Dec 22;15:1273807. doi: 10.3389/fnagi.2023.1273807 (PMC10768723; doi:10.3389/fnagi.2023.1273807)
Supplement: Supplementary file 4 [file Data_Sheet_4.docx]

Supplementary Material

Urine metabolomics phenotyping and urinary biomarker exploratory in mild cognitive impairment and Alzheimer’s disease

Yuye Wang, Yu Sun, Yu Wang, Shuhong Jia, Yanan Qiao, Zhi Zhou, Wen Shao, Xiangfei Zhang, Jing Guo, Xincheng Song, Xiaoqian Niu, Dantao Peng^*^

*** Correspondence:** Dantao Peng: pengdantao2000@163.com

# Supplementary data

## Metabolites identification and differential metabolites

Compared to MCI group, there were 133 significantly differential metabolites between the AD and MCI groups, including 101 upregulated ones and 32 downregulated ones. The expression of the differential metabolites in AD group was displayed as a volcano plot (**supplementary** **Figure 12A**). Detailed differential metabolites were shown in a heatmap in **supplementary Figure 12B**. Z-scores of the top 30 differential metabolites were shown in **supplementary Figure 13C**. Besides, the top 10 regulated metabolites either upregulated or downregulated were displayed in a stem plot (**supplementary Figure 13D**). 1H-indol-3-yl(pyridin-2-yl) methanol was the most upregulated metabolite and Metronidazole-OH was the most downregulated metabolite.

## Correlation between metabolites

Among the top 20 differential metabolites ranged by p-value, many metabolites were correlated with each other, positively. A strong correlation (r>0.7) between metabolites was shown in the chord diagram (**supplementary Figure 13A**). In AD-MCI group, desmethylcitalopram, ethyl 2,3-dioxo-1,2,3,4-tetrahydroquinoline-4-carboxylate and 2-(1,3-dimethyl-1H-pyrazol-5-yl)-1H-isoindole-1,3(2H)-dione were found to be positively correlated with each other. Meanwhile, they were all positively correlated with adenylyl sulfate. Besides, WQH was positively correlated with nortriptyline. The overall correlation heatmap was shown in **supplementary Figure 13B** when the red indicated a positive relationship and the blue indicated a negative relationship.

## Enrichment results of KEGG analysis

According to KEGG enrichment analysis results, caffeine metabolism, mTOR signaling pathway and PI3K-Akt signaling pathway were enriched in AD-MCI group (p<0.05). The relative pathways were shown in **supplementary Figure 14A-C**. Moreover, metabolic network showed the relationship among compounds, pathways, modules, enzymes and reactions to facilitate the presentation of the overall metabolic response. In AD-MCI group, 31 compounds were mapped with the KEGG database while 14 compounds were differential metabolites as indicated by green squares. Galactose metabolism, caffeine metabolism, degradation of flavonoids, biosynthesis of unsaturated fatty acids, ferroptosis and GnRH secretion were enriched as shown in **supplementary Figure 15**.

## Identification of novel diagnostic panel

Based on previous analysis, we extracted differential metabolites plus age and APOE ε4 status to construct the LASSO model. Based on LASSO results, we built SVM classifiers with 10-fold cross-validation to investigate the ideal multivariate signatures that distinguished AD from MCI. After training in training sets, we compared the results of test sets using different kernel functions in SVM. For AD-MCI model, 35 metabolites and age were identified when MSE reached minimum with the value of lambda (min) equaling 0.02694 (**supplementary Figure 16A**). The linear kernel function achieved the highest predictive value with an accuracy of 0.8333. **supplementary Figure 16B and C** showed the ROC curves in training set and test set. The specific metabolites included in the diagnostic panel were shown in **supplementary Table 4**. The evaluation of diagnostic models was shown in **supplementary Table 5**.

# Supplementary Figures and Tables

## Supplementary Fig

##
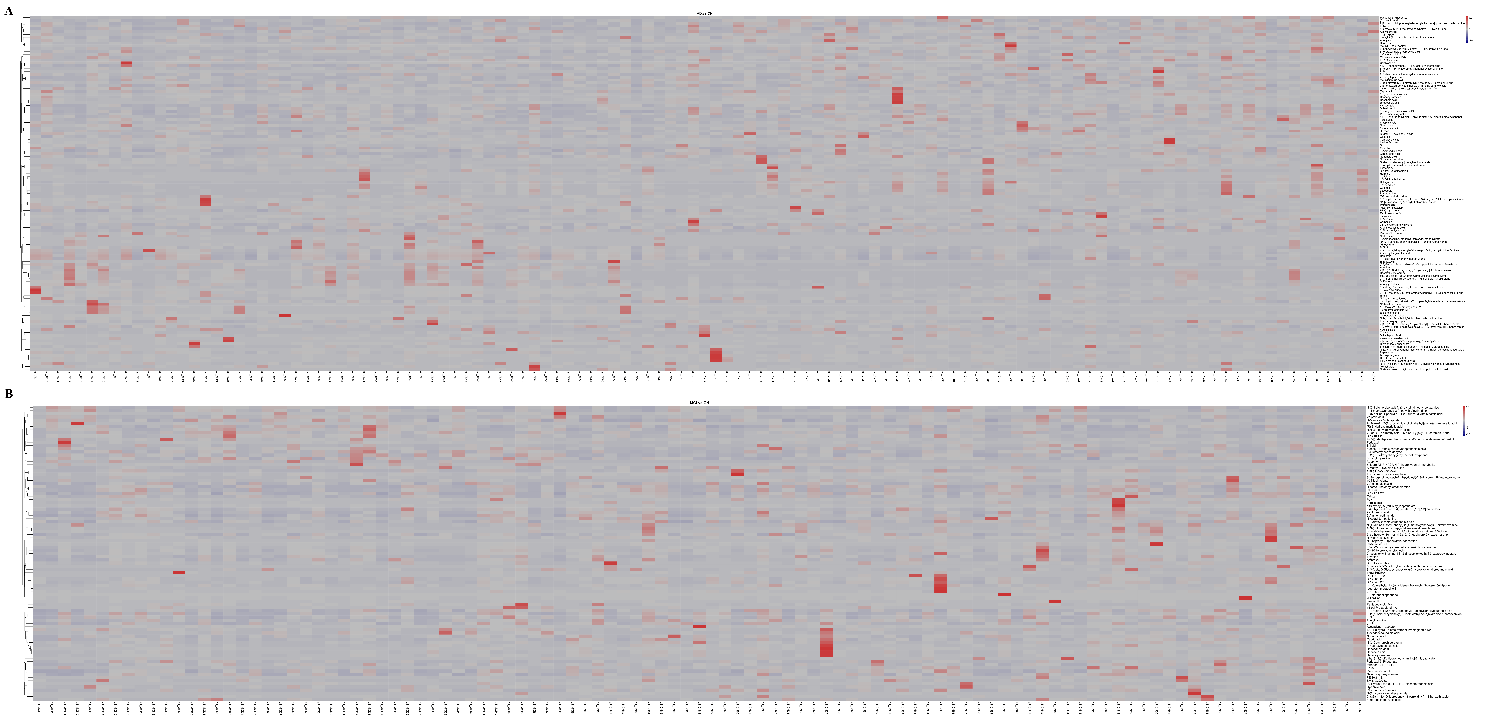
Supplementary Figure 1. Clustered heatmap of all differential metabolites. A. Heatmap of 125 differential metabolites in AD-CN group. B. Heatmap of 93 differential metabolites in MCI-CN group. Red indicates upregulation and blue indicates downregulation.
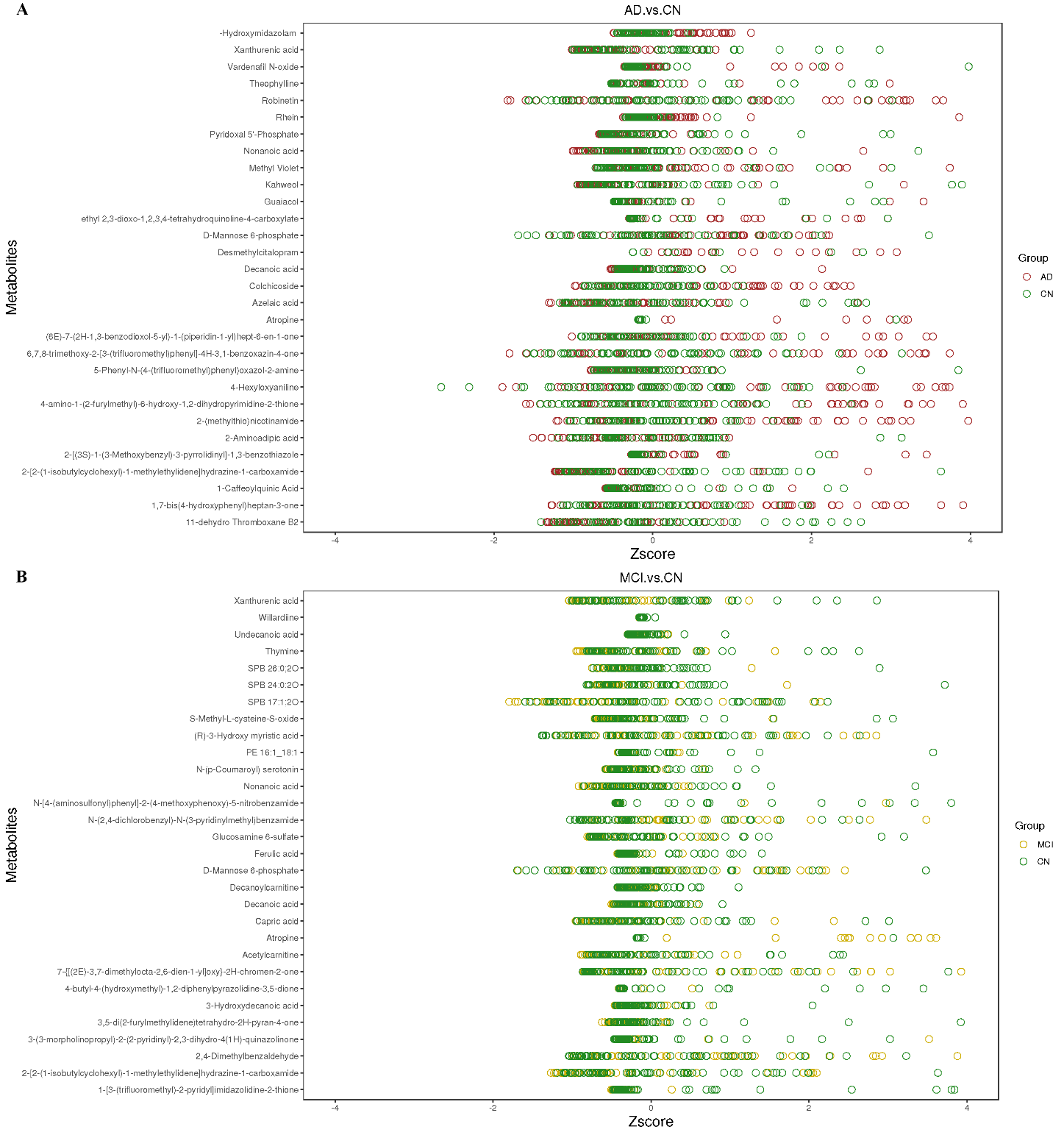
Supplementary Figure 2. Z-score of top 30 differential metabolites ranged by T-test *p*-value. A. Z-score of top 30 differential metabolites in AD-CN group. B. Z-score of top 30 differential metabolites in MCI-CN group. Brown indicates AD samples. Gold indicates MCI samples. Green indicates CN samples.

##
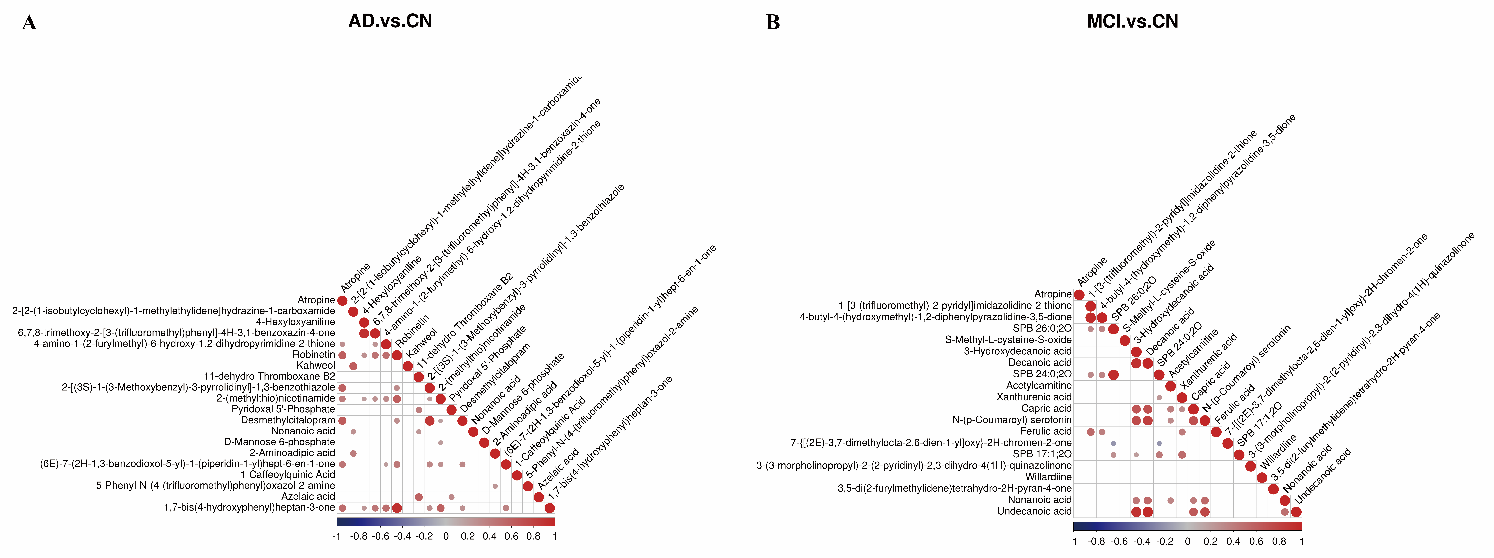
Supplementary Figure 3. Correlation heatmap between top 20 differential metabolites ranged by correlation p-value. A. Correlation heatmap in AD-CN group. B. Correlation heatmap in MCI-CN group. Red indicates positive correlation and blue indicates negative correlation


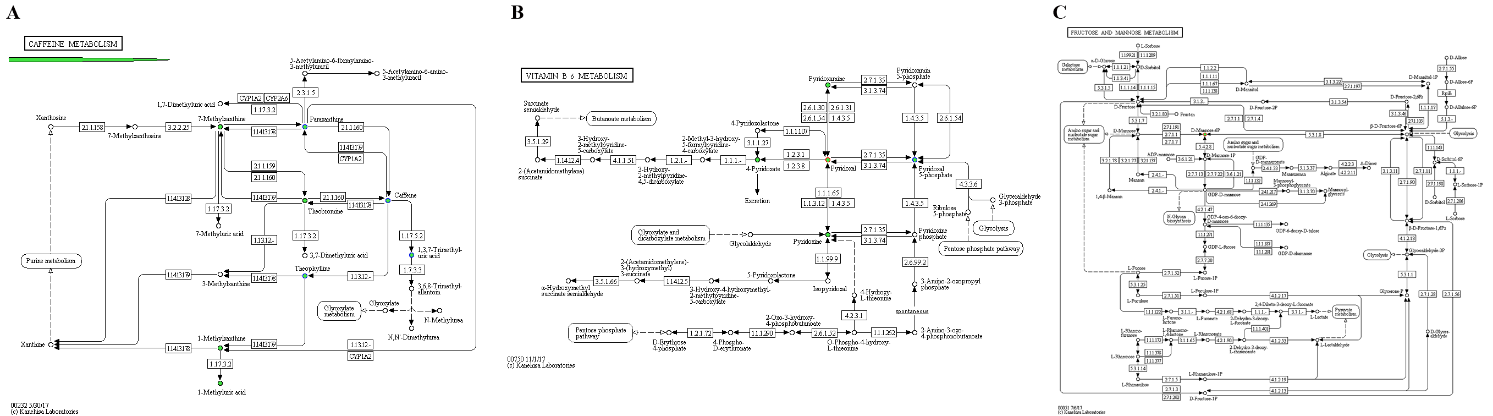
**Supplementary Figure 4.** KEGG pathway enriched by differential metabolites. A. Caffeine metabolism pathway enriched in AD-CN group. B. Vitamin B6 metabolism pathway enriched in MCI-CN group. C. Fructose and mannose metabolism pathway enriched in MCI-CN group. The green dots indicate annotated metabolites. The red edge indicates upregulated differential metabolites. The blue edge indicates downregulated differential metabolites


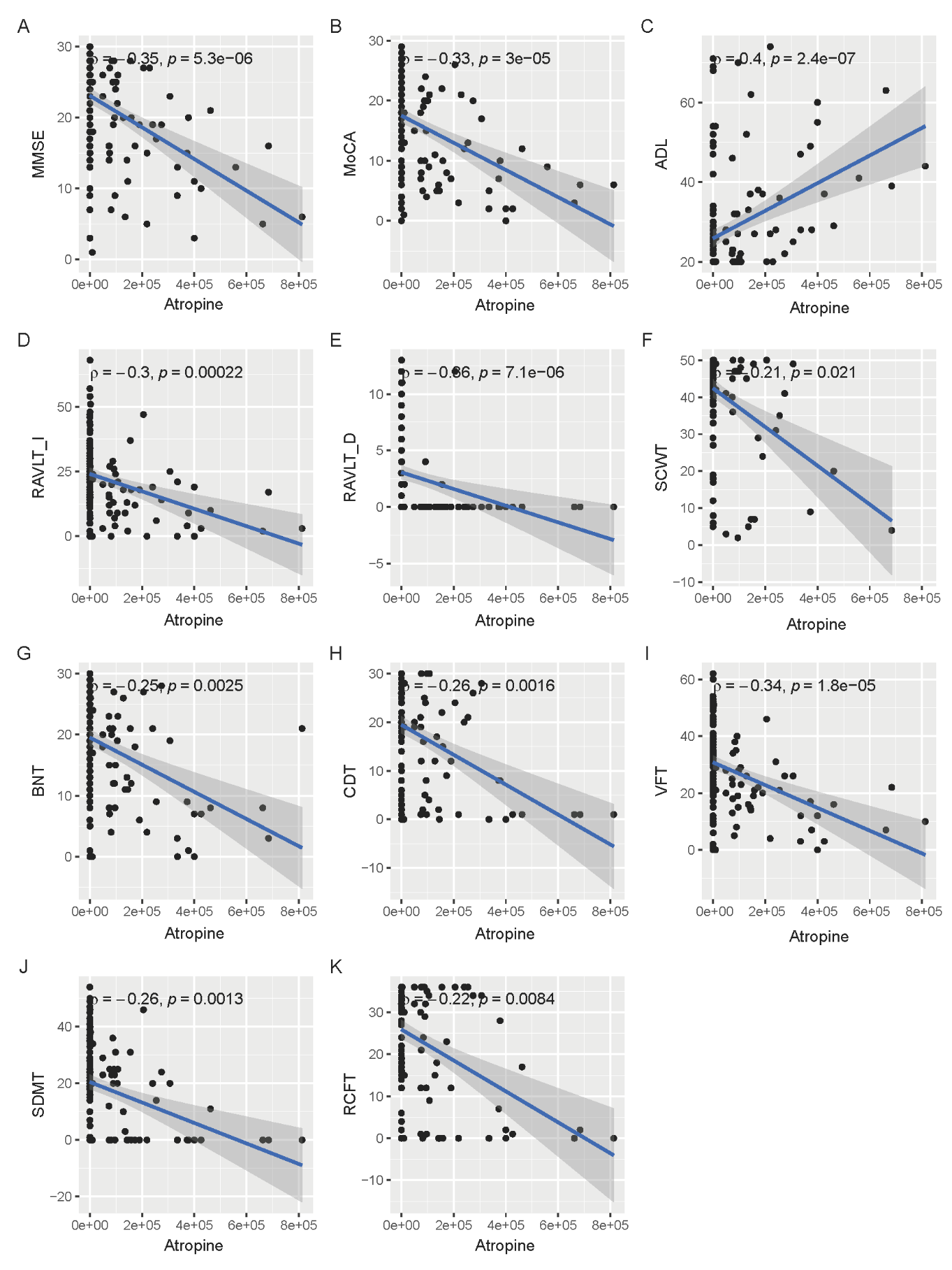


**Supplementary Figure 5.** Scatter plots of atropine with different cognition tests (p<0.05)

**
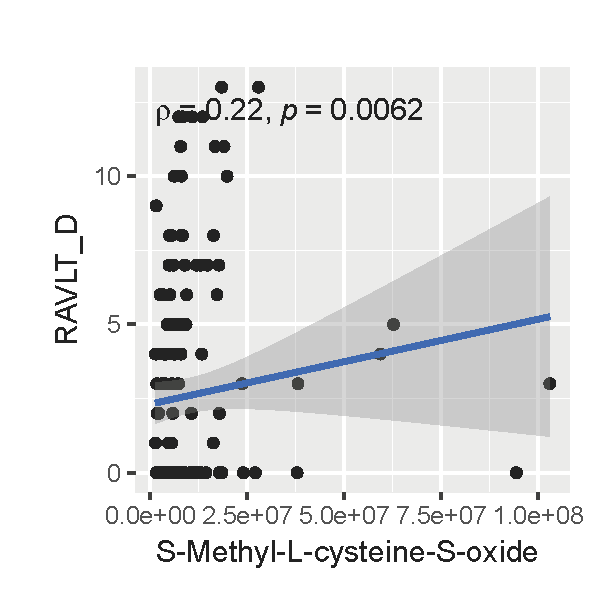
**

**Supplementary Figure 6.** Scatter plots of S-Methyl-L-cysteine-S-oxide with RAVLT_I (p<0.05)


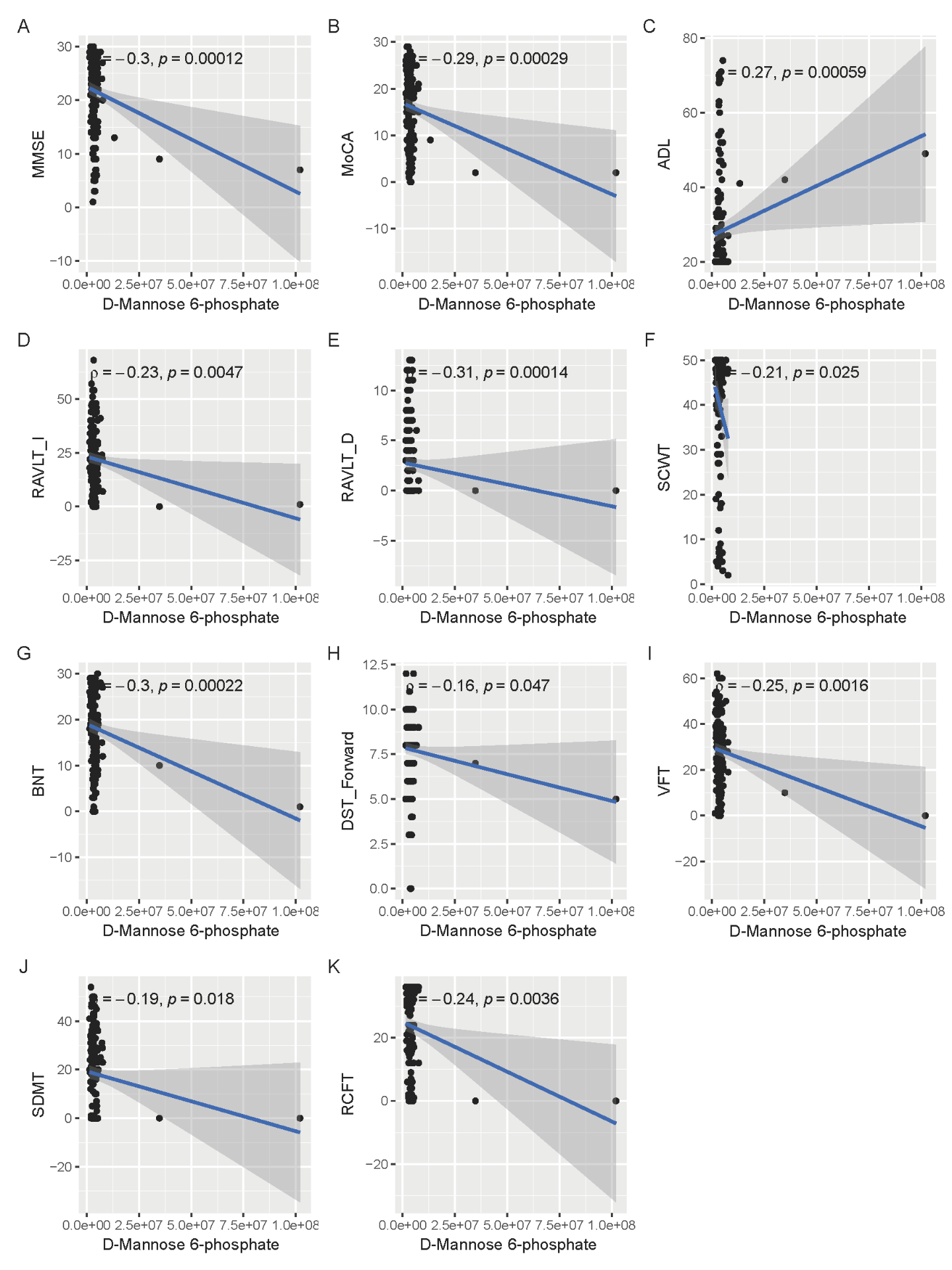


**Supplementary Figure 7.** Scatter plots of D-Mannose 6-phosphate with different cognition tests (p<0.05)


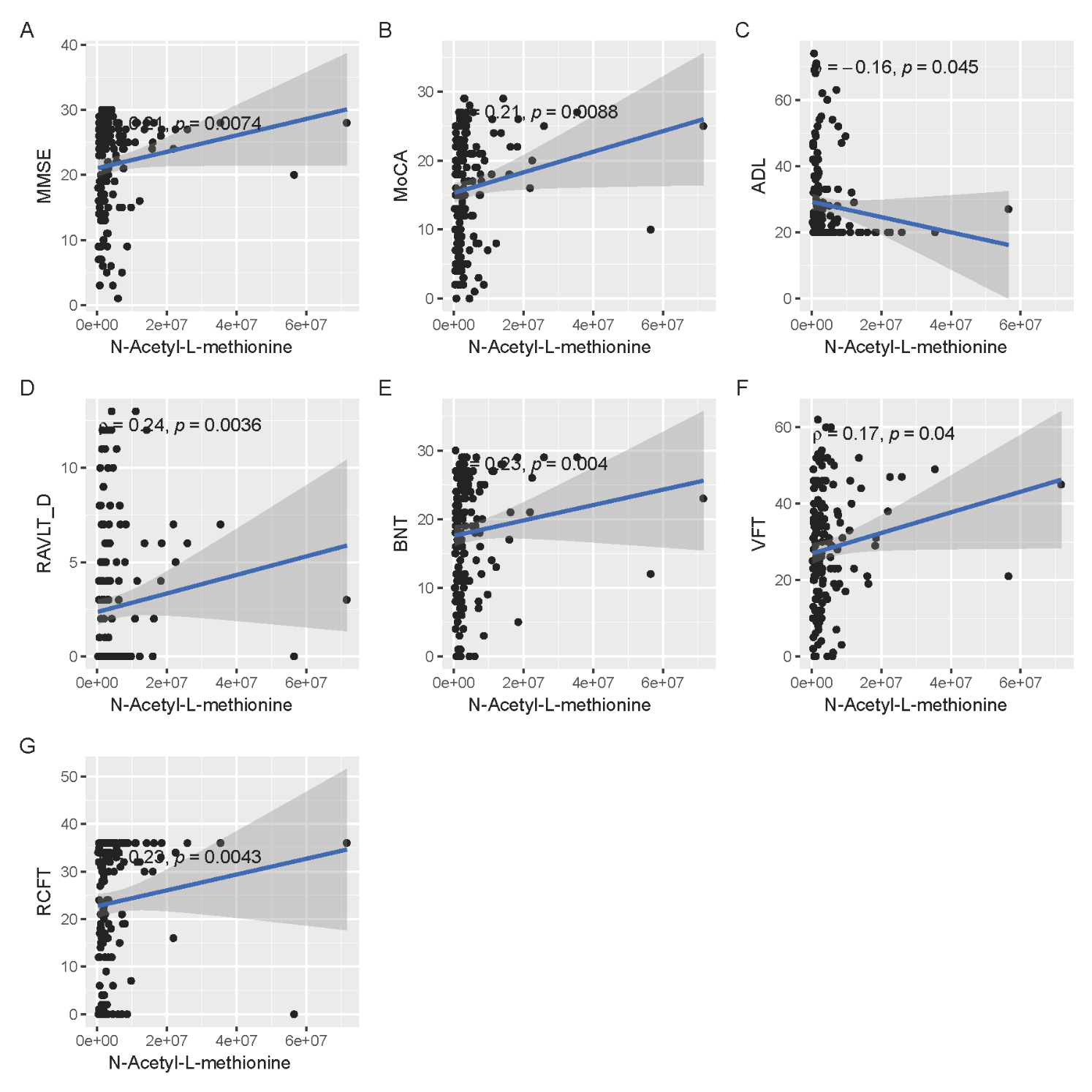


**Supplementary Figure 8.** Scatter plots of N-Acetyl-L-methionine with different cognition tests (p<0.05)


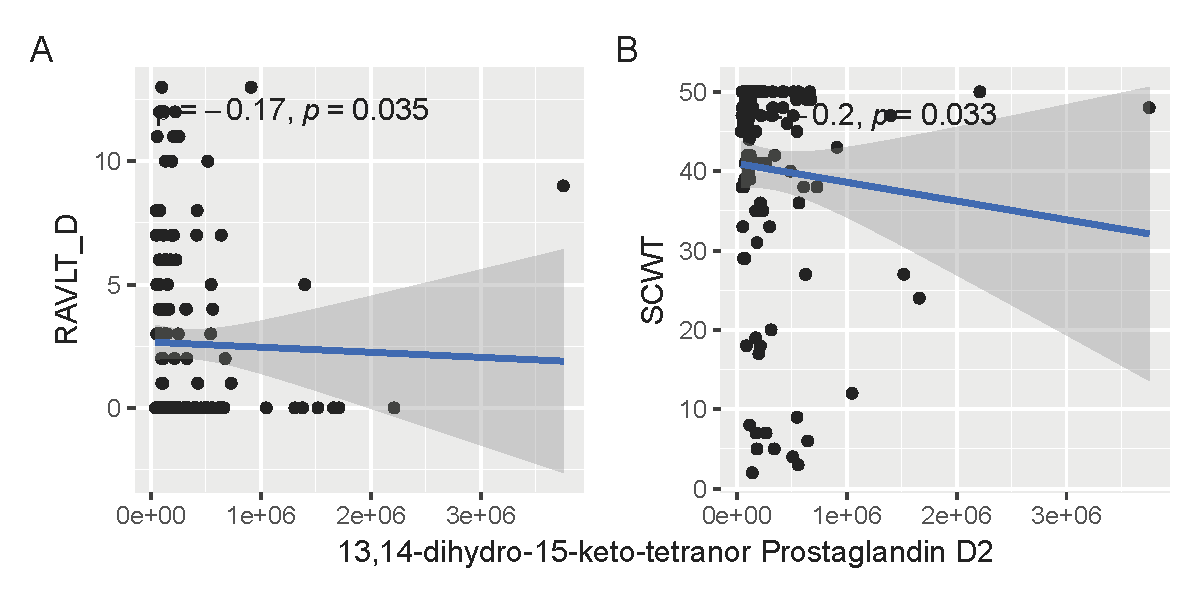


**Supplementary Figure 9.** Scatter plots of 13,14-dihydro-15-keto-tetranor Prostaglandin D2 with different cognition tests (p<0.05)


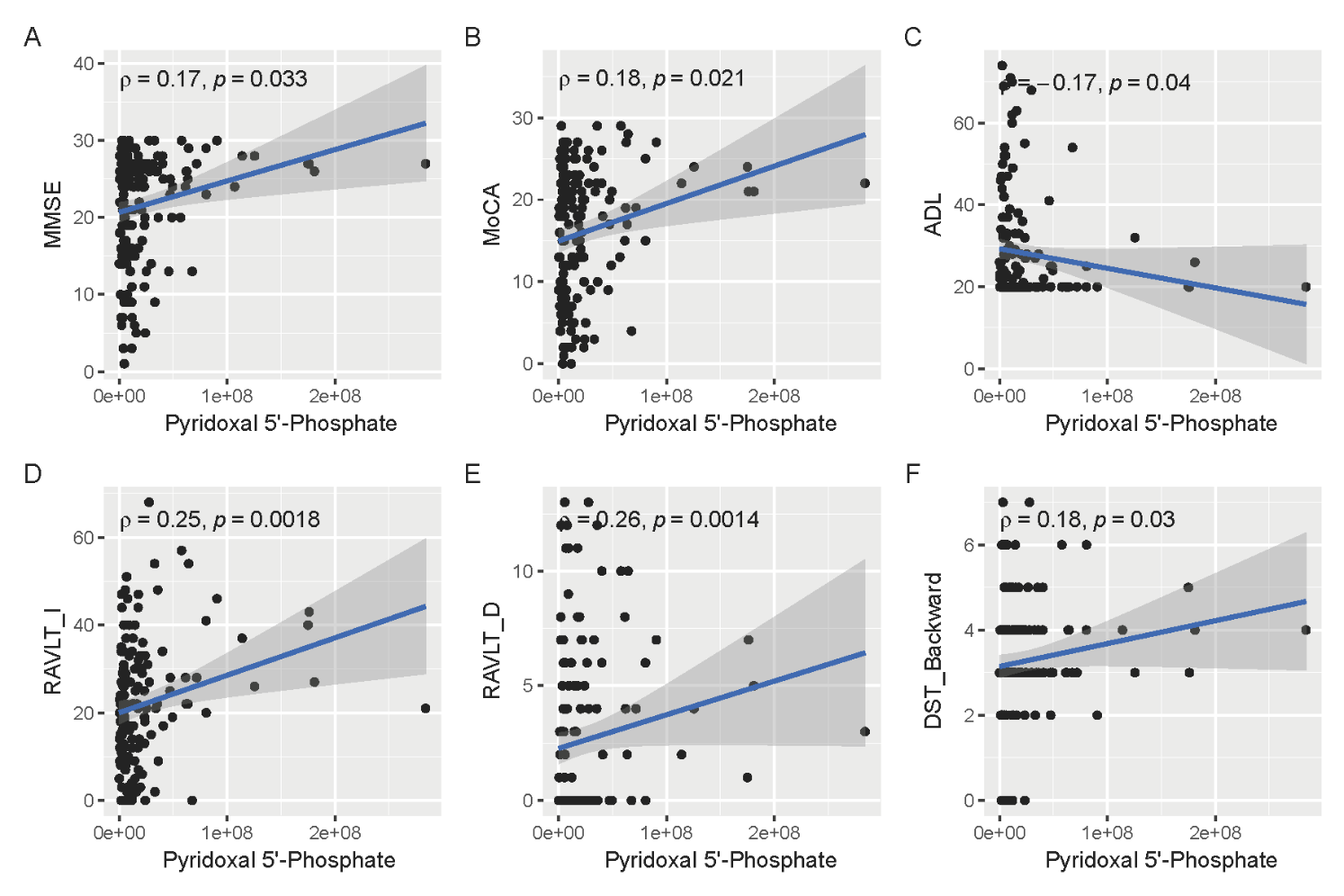


**Supplementary Figure 10.** Scatter plots of Pyridoxal 5'-Phosphate with different cognition tests (p<0.05)


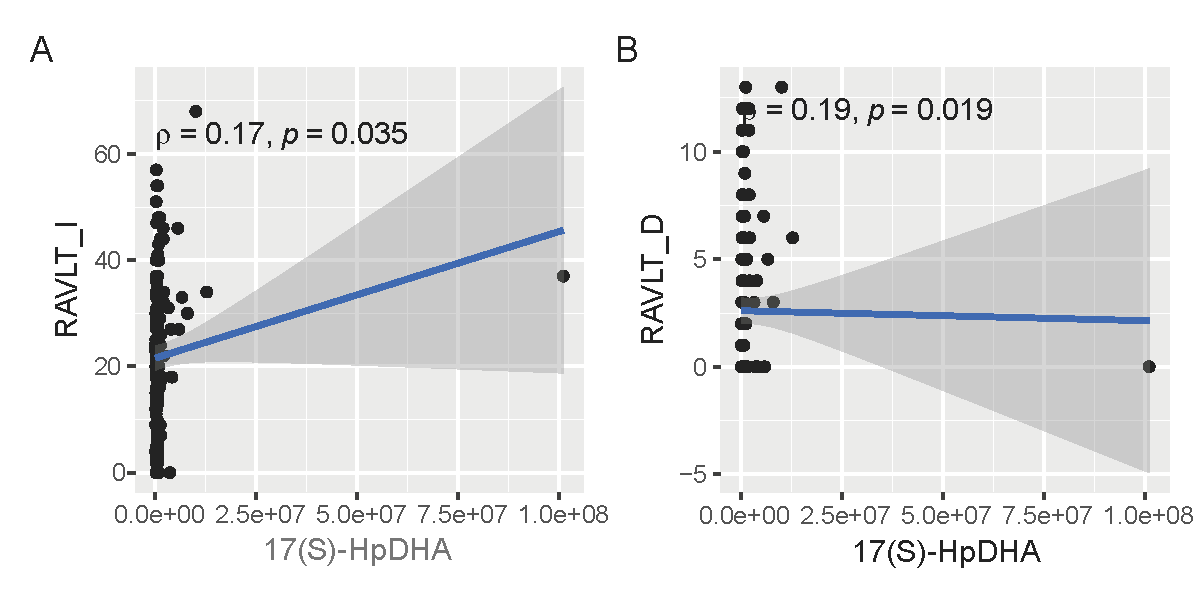


**Supplementary Figure 11.** Scatter plots of 17(S)-HpDHA with different cognition tests (p<0.05)


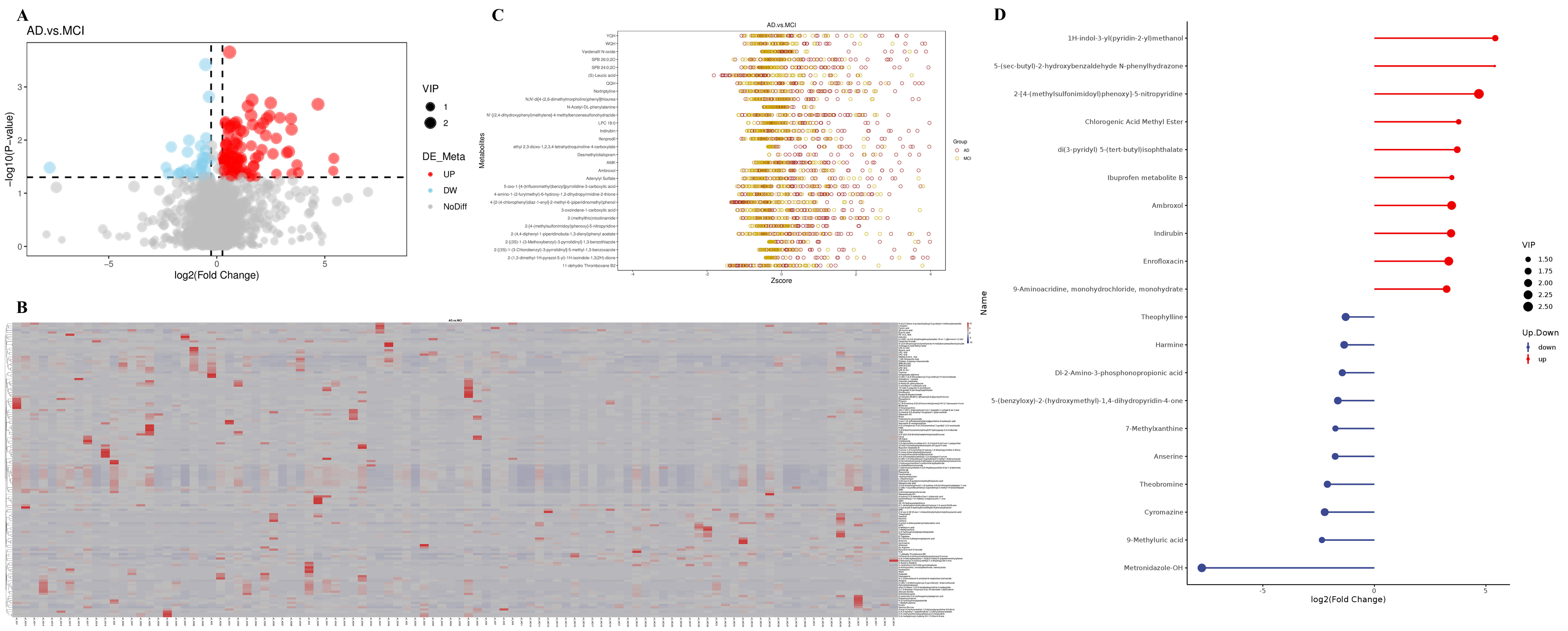


**Supplementary Figure 12.** Differential metabolites and top differential metabolites in AD-MCI group A. Volcano plot showed the distribution of all metabolites between AD and MCI. B. Heatmap of 133 differential metabolites in AD-MCI group. C. Z-score of top 30 differential metabolites ranged by T-test p-value in AD-MCI group. Brown indicates AD samples. Gold indicates MCI samples. D. Stem plot of top 20 differential metabolites in AD-MCI group. Red indicates upregulation and blue indicates downregulation. The size of the circle indicates the VIP value.


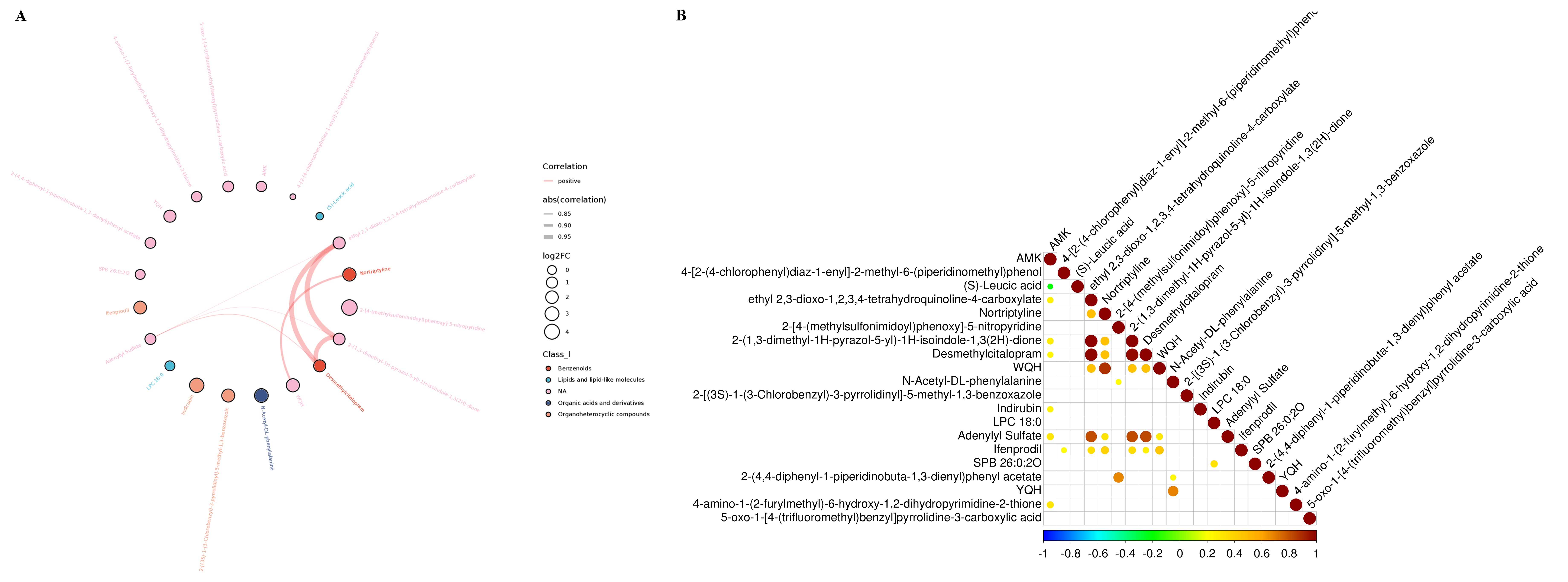


**Supplementary Figure 13.** Correlation between differential metabolites in AD-MCI group. **A**. Chord plots showing the relationship between the top 20 differential metabolites ranged by correlation p-value. The red line indicates a positive correlation. The width of the line indicates the absolute value of the correlation coefficient. The size of the circle indicates log2 fold change. The colors of the circles indicate the classification of metabolites. **B**. Correlation heatmap between top 20 differential metabolites ranged by correlation p-value. Red indicates positive correlation and blue indicates negative correlation.


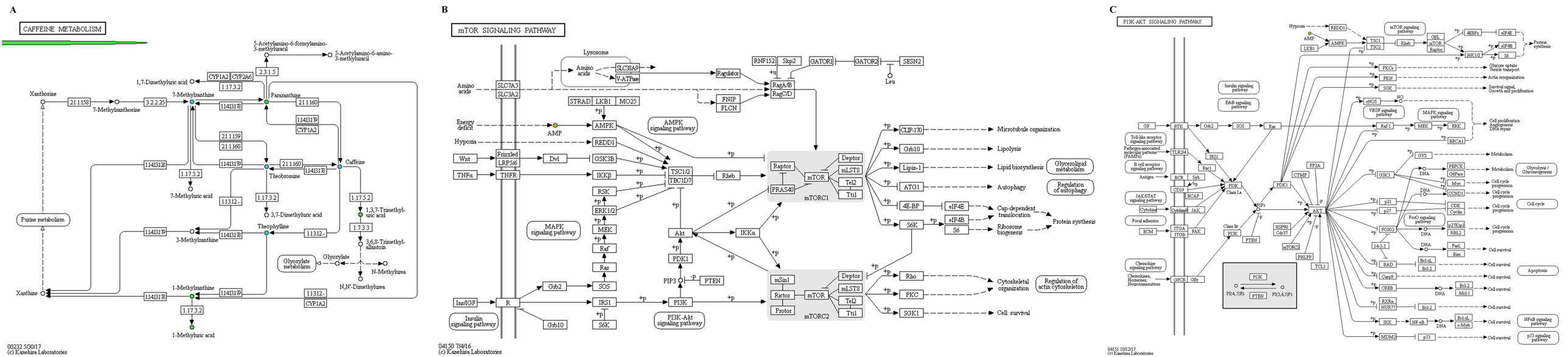


**Supplementary Figure 14.** KEGG pathway enriched by differential metabolites in AD-MCI group. **A**. Caffeine metabolism pathway. **B**. mTOR signaling pathway. **C**. PI3K-AKT signaling pathway. The green dots indicate annotated metabolites. The red edge indicates upregulated differential metabolites. The blue edge indicates downregulated differential metabolites.


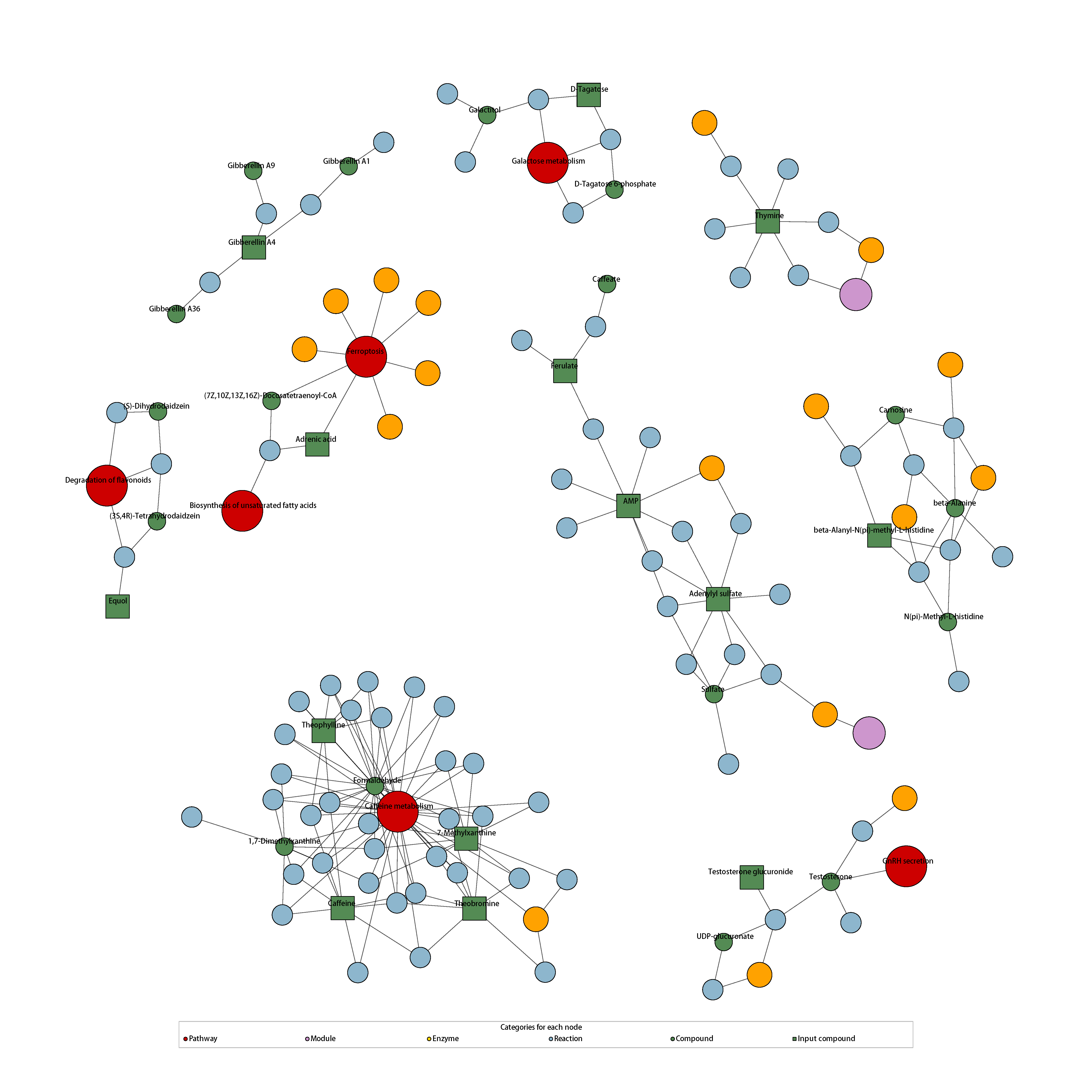


**Supplementary Figure 15.** KEGG regulation map enriched by differential metabolites in AD-MCI group. The red circle indicates a metabolism pathway. The purple circle indicates a module of a class of metabolites. The yellow circle indicates an enzyme related to a certain substance. The blue circle indicates interactions between chemical substances. The green circle indicates background substance in a metabolism pathway. The green square indicates input differential metabolites


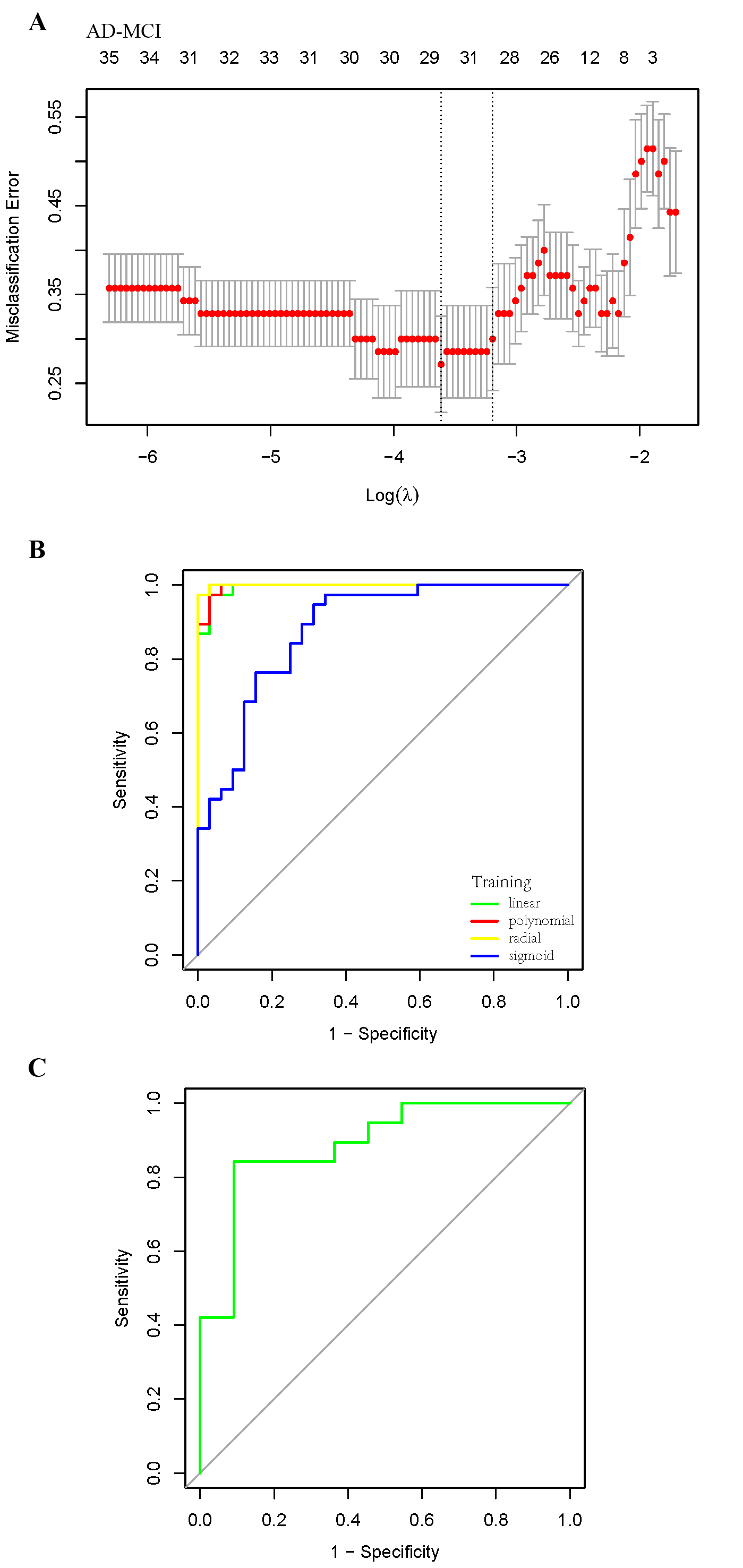


**Supplementary Figure 16.** Diagnostic panel selection and ROC curve constructed by LASSO and SVM in AD-MCI group. A. LASSO model for variable selection. B. ROC curve for AD diagnosis in the training set. C. ROC curve for AD diagnosis in the test set using linear kernel.

## Supplementary Tables

**Supplementary Table 1.** Diagnostic metabolites. Diagnostic metabolites in AD-CN group, MCI-CN group and Hub metabolites are the intersection of two diagnostic panels

| AD-CN | MCI-CN | Hub metabolites |
| --- | --- | --- |
| Atropine | Atropine | Atropine (Com_488_pos) |
| 2-[2-(1-isobutylcyclohexyl)-1-methylethylidene]hydrazine-1-carboxamide | 4-butyl-4-(hydroxymethyl)-1,2-diphenylpyrazolidine-3,5-dione | S-Methyl-L-cysteine-S-oxide (Com_13438_pos) |
| 4-Hexyloxyaniline | S-Methyl-L-cysteine-S-oxide | D-Mannose 6-phosphate (Com_15033_neg) |
| Robinetin | Acetylcarnitine | Spiculisporic Acid (Com_19445_pos) |
| Kahweol | 7-{[(2E)-3,7-dimethylocta-2,6-dien-1-yl]oxy}-2H-chromen-2-one | N-Acetyl-L-methionine (Com_10650_pos) |
| Pyridoxal 5'-Phosphate | Willardiine | 13,14-dihydro-15-keto-tetranor Prostaglandin D2 (Com_30454_neg) |
| D-Mannose 6-phosphate | 3,5-di(2-furylmethylidene)tetrahydro-2H-pyran-4-one | Pyridoxal 5'-Phosphate (Com_2397_neg) |
| 1-Caffeoylquinic Acid | Nonanoic acid | 17(S)-HpDHA (Com_4433_neg) |
| 4-Methylhippuric acid | Thymine |  |
| Omeprazole sulphone | Glucosamine 6-sulfate |  |
| 4-methoxy-6-[2-(4-methoxyphenyl)ethyl]-2H-pyran-2-one | D-Mannose 6-phosphate |  |
| N-Acetyl-L-methionine | PE 16:1_18:1 |  |
| Benzoic acid | (R)-3-Hydroxy myristic acid |  |
| S-Methyl-L-cysteine-S-oxide | N-(2,4-dichlorobenzyl)-N-(3-pyridinylmethyl)benzamide |  |
| L-Anserine | 2,4-Dimethylbenzaldehyde |  |
| N'-[(2,4-dihydroxyphenyl)methylene]-4-methylbenzenesulfonohydrazide | 6-Aminonicotinamide |  |
| 17(S)-HpDHA | Spiculisporic Acid |  |
| 13,14-dihydro-15-keto-tetranor Prostaglandin D2 | 10-Nitrolinoleate |  |
| Etiocholanolone | N-Acetyl-L-methionine |  |
| Flunitrazepam-d3 | Pentadecanoic Acid |  |
| 5-Methyl-8-nitro-3-spirocyclohexyl-2,3,4,5-tetrahydro-1H-2-benzazepine | 13,14-dihydro-15-keto-tetranor Prostaglandin D2 |  |
| P-Coumaroyl Agmatine | 3-[3-(beta-D-Glucopyranosyloxy)-2-hydroxyphenyl]propanoic acid |  |
| Pelargonidin | Pyridoxal 5'-Phosphate |  |
| Sodium [dodecanoyl(methyl)amino]acetate | 3,4-dihydro-2H-benzo[4,5]imidazo[2,1-b][1,3]thiazin-3-ol |  |
| 5-Hydroxyindole-2-carboxylic acid | Avocadyne 1-acetate |  |
| Argininosuccinic acid | 17(S)-HpDHA |  |
| Flavin mononucleotide (FMN) | 4-acetyl-4-(ethoxycarbonyl)heptanedioic acid |  |
| N-{6-[(7-chloro-4-quinazolinyl)oxy]-3-pyridinyl}-4-methoxybenzamide | Thymidine 5'-monophosphate |  |
| 2-Phosphoglyceric acid | 2-[(5-anilino-4-phenyl-4H-1,2,4-triazol-3-yl)thio]acetic acid |  |
| Spiculisporic Acid | Cyclamic acid |  |
|  | 2-Aminoadipic acid |  |
|  | ethyl 3-[3,5-di(trifluoromethyl)anilino]-2-nitroacrylate |  |
|  | 2-Hydroxy-2-methylbutanedioic acid |  |
|  | PE 17:0_18:2 |  |
|  | 2-[(3S)-1-(3,4-Difluorobenzyl)-3-pyrrolidinyl]-1,3-benzothiazole |  |
|  | Valproic acid |  |
|  | 3-[4-methyl-1-(2-methylpropanoyl)-3-oxocyclohexyl]butanoic acid |  |
|  | Pyridoxal |  |
|  | N-lactoyl-phenylalanine |  |
|  | 3-[2-(3-Hydroxyphenyl)ethyl]-5-methoxyphenol |  |
|  | PLH |  |
|  | SM 8:0;2O/26:0 |  |
|  | Ifenprodil |  |
|  | 2-(2-oxo-2-{[2-(2-oxo-1-imidazolidinyl)ethyl]amino}ethoxy)acetic acid |  |
|  | 4-morpholinobenzoic acid |  |

**Supplementary Table 2.** Evaluation of diagnostic value using accuracy, precision, recall, F1-measure and AUC in different SVM models. **A**. Training set; **B**. Test set

1. Training set

| Training set | AD-CN | | | | MCI-CN | | | |
| --- | --- | --- | --- | --- | --- | --- | --- | --- |
|  | linear | polynomial | radial | sigmoid | linear | polynomial | radial | sigmoid |
| Accuracy | 0.9643 | 0.9762 | 0.9881 | 0.9405 | 0.8784 | 1.0000 | 1.0000 | 0.6216 |
| Precision | 0.9512 | 0.9524 | 0.9756 | 1.0000 | 0.9615 | 1.0000 | 1.0000 | 0.5758 |
| Recall | 0.975 | 1.0000 | 1.0000 | 0.8750 | 0.7576 | 1.0000 | 1.0000 | 0.5758 |
| F1-measure | 0.9630 | 0.9756 | 0.9876 | 0.9333 | 0.8475 | 1.0000 | 1.0000 | 0.5758 |
| AUC | 0.9972 | 0.9994 | 1.0000 | 0.9903 | 0.9682 | 1.0000 | 1.0000 | 0.5684 |

1. Test set

| Test set | AD-CN | | | | MCI-CN | | | |
| --- | --- | --- | --- | --- | --- | --- | --- | --- |
|  | linear | polynomial | radial | sigmoid | linear | polynomial | radial | sigmoid |
| Accuracy | **0.9143** | 0.9143 | 0.8 | 0.8 | **0.6452** | 0.6129 | 0.5806 | 0.6129 |
| Precision | **0.9375** | 0.9375 | 1.0000 | 1.0000 | **0.4444** | 0.4286 | 0.2857 | 0.4286 |
| Recall | **0.8824** | 0.8824 | 0.5882 | 0.5882 | **0.4000** | 0.6000 | 0.2000 | 0.6000 |
| F1-measure | **0.9091** | 0.9091 | 0.7407 | 0.7407 | **0.4210** | 0.5000 | 0.2353 | 0.5000 |
| AUC | **0.9575** | 0.902 | 0.9346 | 0.9902 | **0.7333** | 0.7429 | 0.6 | 0.5762 |

**Supplementary Table 3.** Spearman correlation between diagnostic metabolites and cognition tests. Relative correlation coefficient *ρ* and significance *p* (two-sided)

| **Spearman correlation** | | | | | | | | | |
| --- | --- | --- | --- | --- | --- | --- | --- | --- | --- |
|  | | Com_488_pos | Com_13438_pos | Com_15033_neg | Com_19445_pos | Com_10650_pos | Com_30454_neg | Com_2397_neg | Com_4433_neg |
| MMSE | ρ | -.349^**^ | .130 | -.298^**^ | .065 | .210^**^ | -.091 | .167^*^ | .096 |
|  | sig. (two sided) | <0.001 | .099 | <0.001 | .413 | .007 | .251 | .034 | .225 |
| MoCA | ρ | -.326^**^ | .143 | -.286^**^ | .081 | .208^**^ | -.133 | .185^*^ | .132 |
|  | sig. (two sided) | <0.001 | .074 | <0.001 | .310 | .009 | .096 | .021 | .100 |
| ADL | ρ | .403^**^ | -.082 | .274^**^ | -.014 | -.162^*^ | .103 | -.166^*^ | -.090 |
|  | sig. (two sided) | <0.001 | .316 | .001 | .863 | .045 | .206 | .040 | .268 |
| RAVLT_I | ρ | -.297^**^ | .126 | -.230^**^ | .087 | .159 | -.069 | .253^**^ | .172^*^ |
|  | sig. (two sided) | <0.001 | .124 | .005 | .288 | .052 | .401 | .002 | .035 |
| RAVLT_D | ρ | -.358^**^ | .223^**^ | -.306^**^ | .117 | .237^**^ | -.172^*^ | .258^**^ | .192^*^ |
|  | sig. (two sided) | <0.001 | .006 | <0.001 | .152 | .004 | .035 | .001 | .019 |
| SCWT | ρ | -.212^*^ | .114 | -.208^*^ | .153 | .173 | -.196^*^ | .135 | .016 |
|  | sig. (two sided) | .021 | .219 | .024 | .097 | .061 | .033 | .144 | .860 |
| BNT | ρ | -.245^**^ | .105 | -.297^**^ | .116 | .234^**^ | -.149 | .133 | .159 |
|  | sig. (two sided) | .003 | .200 | <0.001 | .158 | .004 | .069 | .104 | .052 |
| DST_Forward | ρ | -.107 | .005 | -.163^*^ | .083 | .079 | -.124 | -.017 | .058 |
|  | sig. (two sided) | .192 | .956 | .046 | .315 | .335 | .132 | .835 | .483 |
| DST_Backward | ρ | -.112 | -.064 | -.011 | -.040 | .050 | -.033 | .178^*^ | .146 |
|  | sig. (two sided) | .174 | .438 | .891 | .626 | .540 | .688 | .030 | .075 |
| CDT | ρ | -.255^**^ | .096 | -.128 | .022 | .134 | -.137 | .102 | .156 |
|  | sig. (two sided) | .002 | .243 | .120 | .787 | .101 | .095 | .213 | .057 |
| VFT | ρ | -.342^**^ | .093 | -.255^**^ | -.002 | .168^*^ | -.117 | .151 | .131 |
|  | sig. (two sided) | <0.001 | .258 | .002 | .977 | .040 | .155 | .064 | .110 |
| SDMT | ρ | -.261^**^ | .019 | -.194^*^ | -.053 | .152 | -.144 | .149 | .124 |
|  | sig. (two sided) | .001 | .817 | .017 | .519 | .064 | .078 | .069 | .131 |
| RCFT | ρ | -.215^**^ | .039 | -.237^**^ | .053 | .232^**^ | -.105 | .125 | .155 |
|  | sig. (two sided) | .008 | .639 | .004 | .524 | .004 | .202 | .127 | .059 |

**Supplementary Table 4.** Diagnostic metabolites in AD-MCI group.

| AD-MCI |
| --- |
| (S)-Leucic acid |
| Nortriptyline |
| N-Acetyl-DL-phenylalanine |
| Indirubin |
| LPC 18:0 |
| Ifenprodil |
| 2-(4,4-diphenyl-1-piperidinobuta-1,3-dienyl)phenyl acetate |
| 2-(methylthio)nicotinamide |
| 11-dehydro Thromboxane B2 |
| 4-acetyl-4-(ethoxycarbonyl)heptanedioic acid |
| 4-(morpholinocarbothioyl)benzonitrile |
| 5-(4-methylphenyl)-2-phenyl-6H-1,3-thiazin-6-one |
| Evernic acid |
| Adenosine 5'-monophosphate |
| Gibberellin A4 |
| Adrenic acid |
| D-Tagatose |
| SPK |
| Atropine |
| 3-(4-hydroxyphenyl)propanohydrazide |
| Metronidazole-OH |
| 2-acetamido-3-(4-methoxyphenyl)propanoic acid |
| Thymine |
| N-{6-[(7-chloro-4-quinazolinyl)oxy]-3-pyridinyl}-4-methoxybenzamide |
| 3-[5-(benzylthio)-4-methyl-4H-1,2,4-triazol-3-yl]-5-oct-1-ynylpyridine |
| 5-(benzyloxy)-2-(hydroxymethyl)-1,4-dihydropyridin-4-one |
| YLK |
| Avocadyne 1-acetate |
| MPH |
| Anserine |
| Cyromazine |
| 4-Hexyloxyaniline |
| 2-chloro-6-(3,5-dimethyl-1H-pyrazol-1-yl)benzonitrile |
| Kinetin |
| (R)-Equol |

**Supplementary Table 5.** Evaluation of diagnostic value using accuracy, precision, recall, F1-measure and AUC in different SVM models. **A**. Training set; **B**. Test set

1. Training set

| Training set | AD-MCI | | | |
| --- | --- | --- | --- | --- |
|  | linear | polynomial | radial | sigmoid |
| Accuracy | 0.9571 | 0.9286 | 0.9857 | 0.8143 |
| Precision | 0.9487 | 0.9714 | 1.0000 | 0.7907 |
| Recall | 0.9737 | 0.8947 | 0.9737 | 0.8947 |
| F1-measure | 0.9610 | 0.9315 | 0.9867 | 0.8395 |
| AUC | 0.9942 | 0.9959 | 0.9992 | 0.8799 |

1. Test set

| Test set | AD-MCI | | | |
| --- | --- | --- | --- | --- |
|  | linear | polynomial | radial | sigmoid |
| Accuracy | **0.8333** | 0.8 | 0.6667 | 0.7667 |
| Precision | **0.8889** | 0.9333 | 0.6957 | 0.8333 |
| Recall | **0.8421** | 0.7368 | 0.8421 | 0.7895 |
| F1-measure | **0.8649** | 0.8235 | 0.7619 | 0.8108 |
| AUC | **0.89** | 0.823 | 0.6986 | 0.8852 |
